# Synthesis, Biological Activity, and Molecular Dynamics Simulations of LNA‐Charge Neutral Linkages for Enhanced Splice‐Switching Antisense Oligonucleotides

**DOI:** 10.1002/anie.202511386

**Published:** 2025-09-21

**Authors:** Alice Kennett, Lillian Lie, Martin Flerin, Belma Zengin Kurt, Ysobel R. Baker, Alyssa C. Hill, Abinaya Ramesh, Matthew J.A. Wood, Debashis Dhara, Afaf H. El‐Sagheer, Fernanda Duarte, Tom Brown

**Affiliations:** ^1^ Department of Chemistry University of Oxford Chemistry Research Laboratory 12 Mansfield Road Oxford OX1 3TA UK; ^2^ Department of Pharmaceutical Chemistry Bezmialem Vakif University Faculty of Pharmacy Istanbul 34093 Türkiye; ^3^ School of Chemistry University of Southampton Highfield Southampton SO17 1BJ UK; ^4^ Department of Paediatrics Institute of Developmental and Regenerative Medicine (IDRM) University of Oxford Oxford OX3 7TY UK

**Keywords:** Antisense oligonucleotide, Gymnotic activity, Locked nucleic acid, Neutral linkage, Splice‐switching

## Abstract

Antisense oligonucleotides are promising therapeutic agents for a range of diseases, having found special clinical success for splice‐switching genetic conditions such as spinal muscular atrophy and Duchenne muscular dystrophy. However, novel chemistries are still required to discover modifications which improve their druggable properties. For in vitro studies, thermal duplex stability, resistance to enzymatic degradation and gymnotic cellular activity are important, and biodistribution, toxicology and potency must be optimised for clinical progression. We investigate the combination of locked nucleic acids (LNA) and charge neutral backbones in chimeric ASOs containing 2′‐O‐methyl sugars and phosphorothioate backbones by evaluating their physical and biological properties. Backbones investigated are LNA‐amide, LNA‐carbamate, LNA‐alkoxyamide, and LNA‐sulfamate. Molecular dynamics simulations of these LNA‐charge neutral backbones were conducted to explore the structural features which determine the experimentally observed thermal duplex stability and conformation. The LNA‐sulfamate linkage is of particular interest, forming very stable duplexes with its RNA target and having comparable gymnotic activity to the previously investigated LNA‐amide, while being synthetically more accessible. Together, our studies indicate that a multi‐faceted approach to expanding the ASO chemical space, using a combination of computational and experimental methods, can build structure‐activity relationships and discover novel promising backbones for future therapeutic use.

## Graphical abstract

Splice‐switching oligonucleotides are used to treat severe genetic conditions including Duchenne Muscular Dystrophy, but new chemistries are urgently required to improve their efficacy. In this work locked nucleic acid (LNA) is coupled to different charge neutral DNA backbones which are studied by structural, thermodynamic, and biological methods. The sulfamate linkage in particular shows great promise.

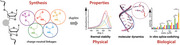

## Introduction

Antisense oligonucleotides (ASOs) have evolved as promising therapeutic agents for targeted gene modulation, and their clinical success marks a transformative era in the field of nucleic acid therapeutics.^[^ ^1^ ^]^ They have gained approval for treating various genetic diseases including Duchenne muscular dystrophy (DMD) and spinal muscular atrophy (SMA), demonstrating their therapeutic potential.^[^ ^2^ ^]^ Ongoing research continues to explore the efficacy of ASOs, siRNAs, and miRNAs^[^ ^3^, ^4^ ^]^ for a wide range of diseases including neurodegenerative disorders, metabolic conditions,^[^ ^5^ ^]^ and cancer.^[^ ^6^ ^]^ Despite their promise, ASOs suffer from poor biodistribution and delivery, reducing their widespread adoption. One of the greatest challenges facing the therapeutic development of ASOs is their inability to efficiently cross cell membranes. For progression into the clinic, an ASO should be able to either passively translocate across the cell membrane or undergo active cellular uptake, endosomal escape, and for some mechanisms of action undergo nuclear localization,^[^ ^7^ ^]^ in order to reach its RNA target.^[^ ^8^ ^]^ Hence, there remains an urgent need for exploring new ASO chemistries with enhanced druggable properties.

Amongst the clinically validated therapeutic oligonucleotide modifications are 2′‐alkylated ribose derivatives^[^ ^9^, ^10^ ^]^ and the phosphorothioate (PS) backbone,^[^ ^11^ ^]^ which in combination, confer increased duplex stability and enhanced enzymatic resistance to the ASO. Charge neutral backbone modifications have been studied in the hope that a reduced overall charge would lower electrostatic repulsion during duplex formation, and that ASOs with increased lipophilicity and reduced anionic charge would cross cell membranes more easily.^[^ ^12^ ^]^ However, many charge neutral backbones, such as amides and carbamates (multiple constitutional isomers), suffer from reduced duplex stability when incorporated between two DNA nucleosides, but do tend to increase enzymatic stability.^[^ ^13^, ^14^, ^15^, ^16^, ^17^ ^]^ The locked nucleic acid (LNA) modification,^[^ ^18^ ^]^ with its rigid bicyclic structure, is a well‐studied modification which enhances RNA binding affinity due to its preferred C3'‐*endo* conformation.^[^ ^19^ ^]^ We previously hypothesized that combining the structural rigidity of LNA with charge neutral linkages would produce ASOs with balanced therapeutic properties. This is indeed the case for the LNA‐amide^[^ ^16^ ^]^ and LNA‐phosphothiotriester.^[^ ^20^ ^]^ An ASO containing four LNA‐amide‐LNA incorporations formed highly stable duplexes, displayed resistance to enzymatic degradation, and most importantly, was more biologically active in a gymnotic splice‐switching reporter assay than the 2′OMe phosphorothioate control ASO.

Building on these promising results, we sought to investigate more synthetically accessible structural variations of the LNA‐amide and probe their biological activity. It is clear by comparing the thermal stability of carbamate backbones to amide backbones that a single atom change can dramatically affect the biophysical properties of the ASO.^[^ ^17^ ^]^ Here, we investigate four distinct neutral backbone modifications in combination with LNA – the amide, carbamate, alkoxyamide, and sulfamate – each linkage offering a unique combination of heteroatoms, linker length, and conformational rigidity. We performed molecular dynamics (MD) simulations of these four backbones to evaluate the stability of the duplex conformations in comparison to an unmodified DNA/RNA duplex and an LNA‐containing DNA/RNA duplex. For the first time, we report the synthesis of LNA‐amide and LNA‐alkoxyamide dimer phosphoramidites. We also synthesized the LNA‐carbamate and LNA‐sulfamate dimer phosphoramidites and incorporated all of these LNA‐charge neutral modifications into ASOs to evaluate their thermal stability and helical conformations. Finally, we tested the biological activity of the ASOs in a splice‐switching reporter assay,^[^ ^21^ ^]^ using both transfection and unassisted gymnotic delivery systems. Using computational, biophysical, and biological data, we aim to establish a better understanding of the structure‐activity relationship for charge neutral heteroatom‐containing linkages. By further investigating the interplay between stability, conformation, and splice‐switching activity, we hope to contribute to ASO design principles and pave the way for the development of more effective therapeutics.

## Results and Discussion

### Synthesis of 5′‐*O*‐DMTr‐Protected Modified Dimer Phosphoramidites

**Figure 1:**
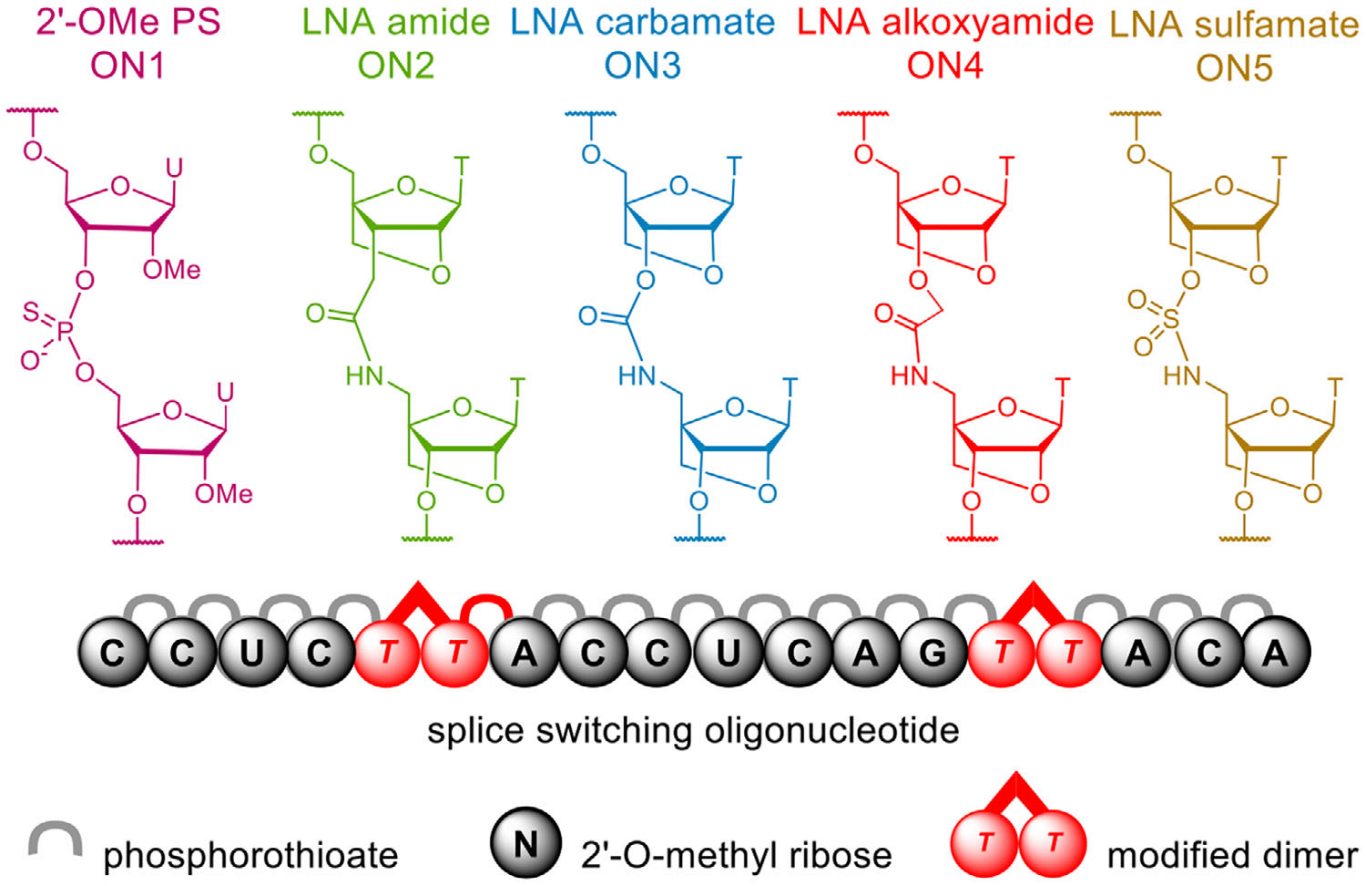
LNA‐charge neutral linkages investigated in a splice switching chimeric oligonucleotide: 2′OMe‐PS control, LNA‐amide, LNA‐carbamate, LNA‐alkoxyamide, and LNA‐sulfamate.

**Scheme 1:**
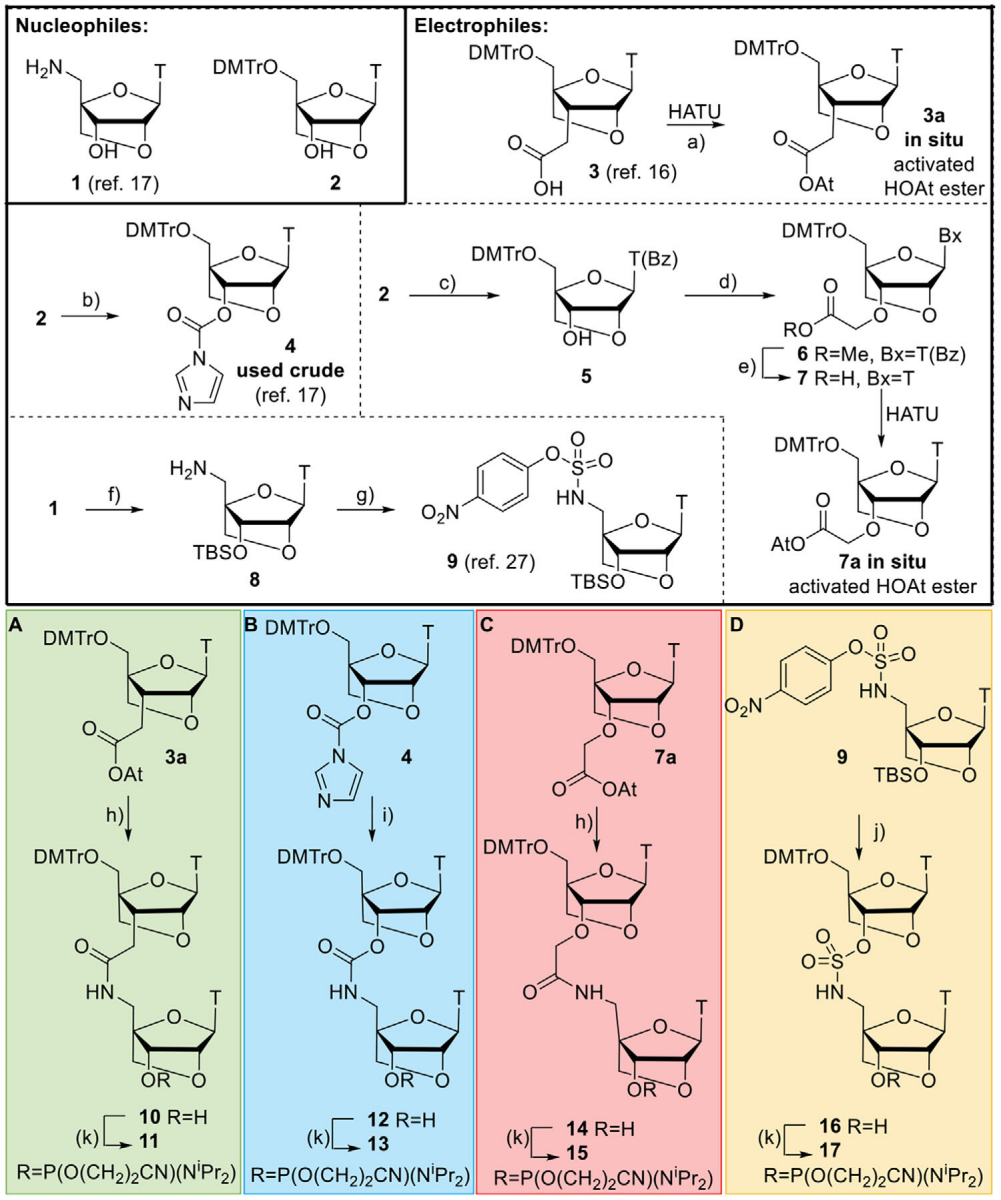
Synthesis of dinucleotide phosphoramidites (**11**, **13**, **15**, **17**): Reagents and conditions: a) HATU, DIPEA, DMF, rt, 30 min, assumed quant.; b) NaH, CDI, THF, 0 °C, 6 h, 79%; c) BSA, MeCN, 80 °C, 1 h, then Et_3_N, BzCl, rt, 16 h, then TBAF (1 M in THF), rt, 5 h, 74%; d) NaH, DMF, 0 °C to rt, 1 h, then methyl bromoacetate, rt, 16 h, 70%; e). 2 M NaOH, THF/MeOH (1:1, v/v), rt, 16 h, 66%; f) (i) MMTrCl, pyridine, rt, 4 h. (ii) TBSCl, imidazole, DMF rt, 16 h. (iii) 3% TCA/CH_2_Cl_2_, rt, 1 h, yield over three steps, 65%, g) 4‐nitrophenol, Et_3_N, CH_2_Cl_2_, 4‐nitrophenylsulfurochloridate, −78 °C, 1 h, then rt, aq. 1 M NaH_2_PO_4_, 49%; h) amine 1, in situ generated HOAt ester, 5 h, rt, **10** 77%, **14** 72%; i) crude 4, DMAP, pyridine, 80 °C, 24 h, 34%; j) DMAP, THF, rt, 16 h, then TBAF (1 M in THF), rt, 5 h, 35%; k) chloro(diisopropylamino)‐β‐cyanoethoxyphosphine, Et_3_N, CH_2_Cl_2_, rt, 3 h, **11** 52%, **13** 57%, **15** 65%, and **17** 65%. **A** is the amide linkage, **B** is carbamate, **C** is alkoxyamide, and **D** is sulfamate. Abbreviations: DMTr 4,4′‐dimethoxytrityl, BSA (*N,O*‐bis(trimethylsilyl)acetamide, HATU hexafluoro‐phosphate azabenzotriazole tetramethyl uranium, HOAt 1‐Hydroxy‐7‐azabenzotriazole, TBS *tert*‐butyldimethylsilyl.

Previously we have shown that the incorporation of four LNA‐amide linkages (Figure 1) in splice‐switching oligonucleotides leads to increased gymnotic activity in cells.^[^ ^16^ ^]^ For oligonucleotide (ON) assembly the automated on‐resin amide coupling can be carried out on the same solid‐phase synthesiser as phosphoramidite chemistry for therapeutic oligonucleotide development.^[^ ^22^ ^]^ Here we carried out ON synthesis using an established dimer phosphoramidite approach to simplify the oligonucleotide construction chemistry.^[^ ^23^ ^]^ With this in mind, amide dinucleotide **10** was synthesized by HATU‐mediated ligation of acid **3** ^[^ ^16^ ^]^ and amine **1** in 77% yield (Scheme 1). This compound was then converted into phosphoramidite **11** in 52% yield (Scheme 1a, green). To investigate the combined effects of the LNA‐charge neutral linkages in 2′OMe/PS therapeutic oligonucleotides on improved cellular uptake we set out to prepare alternative linkages, related to the amide, that are synthetically more accessible.

The LNA‐carbamate linkage (as shown in ON3, Figure 1) is much easier to incorporate into oligonucleotides than the LNA‐amide via synthesis of dinucleotide phosphoramidite **13**, as electrophile **4** can be accessed from commercially available alcohol **2**, while electrophile **3a** (generated in situ as an activated ‐OAt ester) requires a multi‐step synthesis.^[^ ^16^ ^]^ In preliminary investigations the LNA‐carbamate was found to exert a slight negative effect on duplex stability in DNA oligomers (change in duplex melting temperature (Δ*T*_m_)) = −1.5 °C against an RNA target compared to the unmodified DNA control).^[^ ^17^ ^]^ Furthermore, when the LNA‐carbamate was incorporated into oligonucleotides at three sites, there was only a duplex destabilisation of 2.0 °C, indicating that the destabilisation effect caused by the LNA‐carbamate is modest. Encouraged by this, the T‐T LNA‐carbamate dinucleotide phosphoramidite **13** was prepared and subsequently incorporated into 2′OMe/PS oligonucleotides for the first time to assess its potential therapeutic properties. Compound **12** was synthesized following previously reported conditions by reaction of a 3′‐activated imidazole carbamate (used crude from reaction of alcohol **2** with CDI) and amine **1**.^[^ ^17^ ^]^ Phosphitylation of **12** was achieved under standard conditions by reaction with chloro(diisopropylamino)‐β‐cyanoethoxyphosphine to give **13** in 57% yield (Scheme 1b, blue).^[^ ^17^ ^]^

Next, we reasoned that the duplex stability of the alkoxy‐amide linkage could be increased by addition of the two flanking LNA sugars (as shown in ON4, Figure 1). This linkage has been investigated in the context of RNA‐binding, and despite having a one atom‐extension compared to the natural phosphodiester, this amide backbone is only minimally destabilising.^[^ ^24^ ^]^ This linkage has not been reported in the context of therapeutic oligonucleotides. It was particularly interesting to us, being a structural hybrid of LNA‐amide and LNA‐carbamate, as it combines both the 3′‐*O*‐ and the methylene linker. It was envisioned that the 3′‐OCH_2_CO_2_H function could be installed via alkylation of the 3′‐hydroxyl group of alcohol **2** with methyl bromoacetate followed by ester hydrolysis. It is well documented that if performed on N^3^ unprotected thymidine, alkylation occurs primarily at this position,^[^ ^25^ ^]^ and therefore our synthesis began with the N^3^‐benzoylation of **2** following a protocol described for the N^3^‐protection of DMT‐thymidine.^[^ ^26^ ^]^ This protocol utilises a one‐pot, three‐step reaction that includes the temporary protection and deprotection of the 3′‐OH with a trimethylsilyl group to achieve a high yield of mono‐benzoylated **5** (74%). Subsequent alkylation of **5** using NaH and methyl bromoacetate in DMF gave ester **6** in 70% yield. Finally, simultaneous hydrolysis of the thymine N^3^‐benzoyl amide and the 3′‐*O*‐methyl ester by reaction with aqueous sodium hydroxide gave LNA‐T‐3′‐oxyacetic acid **7** in 66% yield. Reaction of **7** with **1** was achieved using a HATU‐mediated amide coupling, which proceeded to give dimer **14** in good yield (72%). Finally, phosphitylation of the dinucleotide **14** was achieved using standard phosphitylation conditions to give phosphoramidite **15** in 65% yield (Scheme 1c, red).

Sulfur‐containing linkages such as the thioamide (5′‐CH_2_SONHCH_2_‐3′), thiocarbamate (5′‐OC (= S)NHCH_2_‐3′), sulfonamide linkage (5′‐CH_2_SO_2_NHCH_2_‐3′), and the sulfamate (5′‐OSO_2_NHCH_2_)‐3′ were considered for investigation as neutral backbones which may have significantly different conformational properties than analogous oxygen‐containing structures (Scheme S1). Direct conversion of amide dinucleotide **10** to the corresponding thioamide using Lawesson's reagent was unsuccessful; this result is supported by later literature, in which a thioamide linkage between RNA nucleosides via a 3′‐thio‐carboxylic acid was synthesised.^[^ ^27^ ^]^ In contrast, the synthesis of a thiocarbamate dimer phosphoramidite was successful, but the oligonucleotide could not be isolated due to instability. This was not investigated further due to reports which suggest that thiocarbonyl linkages are incompatible with ammonia deprotection conditions.^[^ ^28^ ^]^ Finally, the sulfonamide linkage was considered, having been reported between DNA sugars for use in therapeutic oligonucleotides.^[^ ^29^ ^]^ However, the synthetic route required for an LNA thymine sulfonamide dinucleotide was lengthy and did not satisfy our desire to find an easily accessible backbone modification. Therefore, we investigated the sulfamate (as shown in ON5, Figure 1), as this linkage can also be prepared from commercially available alcohol **2** in only a few steps. The LNA‐sulfamate dimer phosphoramidite **17** was synthesized via a 5′‐(4‐nitrophenyl)‐activated sulfamate intermediate **9**. Reaction of this intermediate with alcohol **2**, followed by TBAF‐mediated TBS deprotection gave **16** in 35% yield. Finally, phosphitylation of alcohol **16** under standard conditions gave phosphoramidite dinucleotide **17** in 65% yield (Scheme 1d, yellow).^[^ ^30^ ^]^

### Molecular Dynamics (MD) Simulations

**Figure 2:**
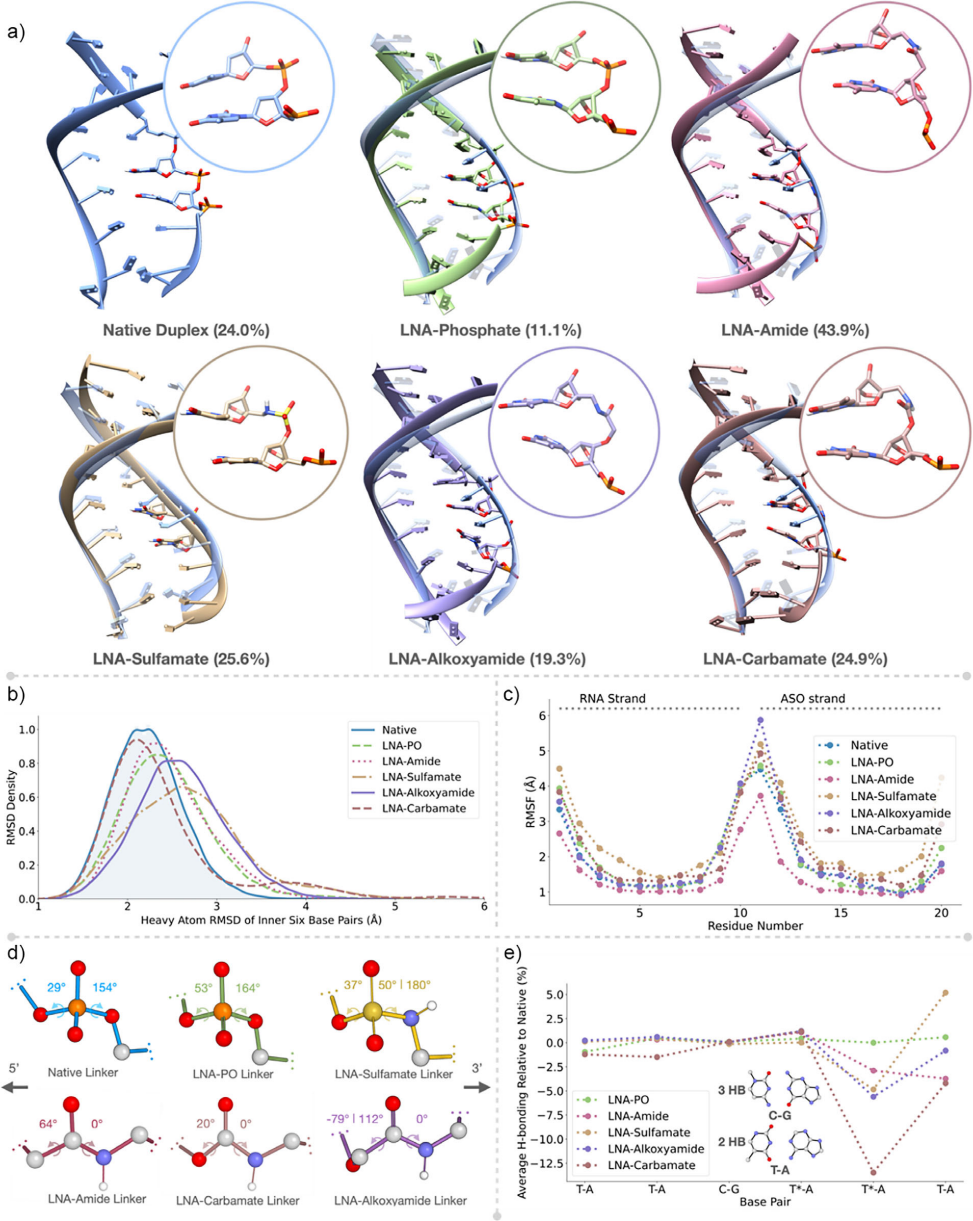
Analysis of 3 µs of Molecular dynamics (MD) simulations of LNA‐charge neutral linkages. a) Representative structure of the most populated cluster for duplexes containing different linkages (left to right): Native phosphate, LNA‐phosphate, LNA‐amide, LNA‐sulfamate, LNA‐alkoxyamide, LNA‐carbamate, overlayed with a representative structure of the most populated cluster of the native phosphate strand for comparison. Values in parentheses indicate the % population of the most populated cluster displayed, following clustering in Chimera. b) Histogram displaying the RMSD distributions of the inner six base pairs of the duplexes with different linkages. c) RMSF values of the residues along the duplex for each system. Horizontal dotted lines indicate the residues of the RNA strand and the ASO strand. d) Comparison of dihedral angles of all linkers throughout the simulation, with values indicating the average of significantly populated angles (more than 20% of simulation time). e) H‐bonding expressed as a percentage of simulation time, between all inner six base pairs, relative to the native strand, for modified systems (see Supporting Information analysis and visualisation section for details).

MD simulations were performed to investigate the impact of locked sugars and neutral linkages on the structure and stability of a model 10‐mer heteroduplex. Attempts to obtain the crystal structure for the all‐2′OMe‐phosphorothioates have previously been unsuccessful, leading us to use the published crystal structure of the d‐CTTTTCTTTG/rCAAAGAAAAG heteroduplex in our study. This structure, with only phosphodiester linkages, was used as a starting point for all simulations and as the control for the unmodified state (Figure 2a, Native Duplex).^[^ ^16^, ^31^ ^]^ Using this sequence, a system containing two locked sugars without modified linkers was generated by manually altering the sugars of the underlined thymidine residues to enforce a C3’‐*endo* conformation (Figure 2a, LNA‐phosphate). Subsequently, the linkers between the two underlined thymidines were modified to produce the LNA‐amide, ‐sulfamate, ‐carbamate, and ‐alkoxyamide starting structures. The modified backbones were positioned sufficiently far from the termini of the duplex to minimise fraying effects on the two T‐A base pairs around them. MD simulations were conducted using the parm99 Amber force field, with LNA parameters obtained from the modified LNA_parm99 force field reported by Condon et al., which account for the pucker of locked nucleotides and the dihedral angle between the sugar and base.^[^ ^32^, ^33^ ^]^ General Amber Force Field (GAFF) parameters were used to model the neutral linkers.^[^ ^34^ ^]^ Each system underwent 3 µs of MD simulation at 300 K and 1 bar (full details in Supporting Information computational methods section). Comparative analysis of the different systems was performed by evaluating several structural parameters. These included average structures, root mean square deviations (RMSDs) and fluctuations (RMSF) of the inner six base pairs, modified linker dihedral angles and distances, and H‐bonding between different inner base pairs during the simulations.

Visual inspection of the most populated cluster structures (Figure 2a) indicates that all systems remain in stable duplexes throughout the simulations (computational methods, Figures S8–S19). This observation is confirmed by comparing the inner base pair RMSD distributions of the modified structures relative to the native strand (Figure 2b). The focus on inner base pairs is intentional, as fraying of duplex ends is expected, and is evident in the RMSF values of the terminal residues (Figure 2c). This is a reversible process on the time‐scale of several microseconds, which can be difficult to study without significantly longer simulation times.^[^ ^35^ ^]^ The deviation of the heavy atoms on the inner six base pairs of the modified duplexes (Figure 2b) is similar to the native system, suggesting that the modifications to the linkers result in only minor distortions to the overall structure. In stark contrast, oligonucleotides modified with backbone alkyl linkers or acyclic sugar analogues have shown significant distortion, with average RMSD values exceeding 5 Å.^[^ ^36^, ^37^ ^]^ The RMSF analysis also indicates that the stabilities of all modified ASOs are comparable with the Native system, with the exception of LNA‐Amide, which displays consistently lower RMSF, suggesting increased stability. Besides the expected terminal fraying, larger jumps in the RMSD over some of the trajectories are attributed to base flipping close to the modified nucleotides (Figure S20).

The main changes resulting from the LNA modifications are observed at the nucleotide level, with the modified linkers assuming different shapes than the native phosphate. A comparison of the dihedral angles of the bonds around each linker central atom shows that the sulfamate is the only one of the linkers, which can adopt similar dihedral angles to the phosphate‐containing native and LNA‐PO systems on the 3′ side of the linker (Figures 2d and S21). On the 5′ side, the dihedral angles of the alkoxyamide are the most distorted with respect to the phosphate‐linked systems, which is a consequence of the linker being one atom longer than the others. The distribution of dihedral angles also affects the distance between C3 and C5 of the sugars around the modified systems (Figure S22), with the sulfamate having the closest average distance to the native and LNA‐PO systems. Additionally, the higher standard deviation (SD, 0.32 Å) of distances observed with the sulfamate is in line with the ability of the linker to populate two different dihedral angles on the 3′ end of the linker. This suggests that the higher flexibility of this neutral linker makes it more similar to the phosphate in its end‐to‐end distance and angle geometries. Although most of the modified linkers show some overlap in the distance distribution with the native system, the carbamate linker has the smallest standard deviation in the distance distribution, which is likely due to its high degree of planarity from conjugation, as evidenced by the dihedral angles. While both the carbamate and the amide have the α‐nitrogen substituent in plane with the carbonyl, the amide 5′ α‐carbon side has a median dihedral angle of 64°, while for the carbamate, the 5′ α‐oxygen side has a median dihedral angle of only 20°.

To quantify the predicted effect of the modifications on H‐bonding, the trajectories were analysed with CPPTRAJ using default hydrogen bonding criteria for the inner six base pairs and are presented in Figure 2f relative to the percentage of H‐bonding in the native control (Supporting Information analysis and visualisation section).^[^ ^38^ ^]^ The locked sugars alone do not disrupt hydrogen bonding compared to the native strand. However, the effect of H‐bonding disruption for charge neutral linker systems is most pronounced at the 5′ thymidine of the two A‐T base pairs around the modified linker site, as well as its upstream unmodified neighbour. The carbamate disrupts H‐bonding by up to 13%, possibly due to its rigidity, while the disruption within the other three modified linkers is comparable at the modified 5′‐thymidine, with suggestions of a slight stabilisation of the unmodified neighbour by the sulfamate. In general, the structures also show evidence of cross‐strand bifurcated H‐bonds which are known to exist in AT‐tracts.^[^ ^39^ ^]^

## Oligonucleotide Synthesis and Physical Properties

**Figure 3:**
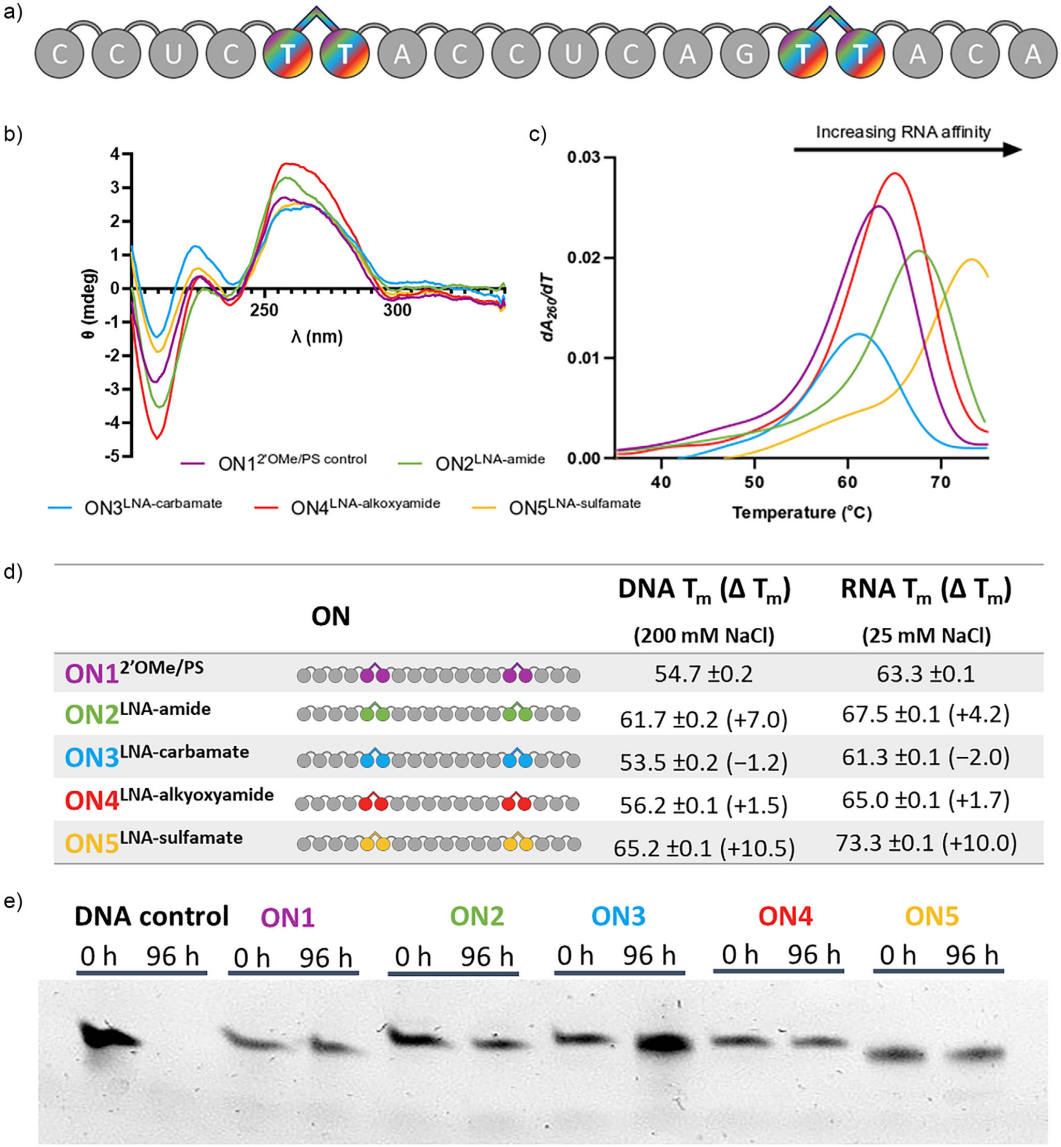
Physical properties of oligonucleotides containing LNA‐charge neutral linkages. a) Sequence of the splice‐switching ASO used in this study; grey circles = 2′OMe nucleotides, rounded linkages = PS, rainbow dimers = LNA‐charge neutral linkages explored (ON2‐5); b) Circular dichroism spectra of ON1‐5 against complementary RNA (10 mM phosphate, 25 mM NaCl, pH = 7.0), *y*‐axis is ellipticity θ in mdeg (10^−3^ deg.cm^2^ dmol^−1^); c) UV‐melting curve derivatives of the ON‐RNA heteroduplexes. The *y*‐axis is derivative of change in UV absorbance at 260 nm; d) Melting temperatures (*T*_m_) of ON1‐5 against complementary RNA (10 mM phosphate, 25 mM NaCl, pH = 7.0), e) Nuclease resistance of ON1‐5 and a DNA phosphate control oligonucleotide against human serum (96 h, 37 °C).

Oligonucleotides ON1‐5 (Figure 3d) were prepared via standard solid phase synthesis using phosphoramidites **11**, **13**, **15** and **17** (Scheme 1). In order to compare the LNA‐charge neutral linkages, we synthesized splice‐switching oligonucleotides whose sequences restore the pre‐mRNA splicing to produce functional luciferase protein in the HeLa pLuc/705 cell line (sequence shown in Figure 3a).^[^ ^40^ ^]^ All phosphoramidites coupled successfully according to the trityl monitor on the ABI‐394 DNA synthesizer and all oligonucleotides were deprotected under standard oligonucleotide deprotection conditions of heating in concentrated aqueous ammonia at 55 °C for 5 h.

We compared the duplex denaturation temperatures (*T*_m_) of the ASOs containing LNA‐charge neutral linkages with that of the 2′OMe/PS control oligonucleotide after hybridisation with DNA and RNA complementary strands (Figure 3c,d). The 2′OMe/PS control was synthesised using commercially available 2′OMe‐U monomers whereas the modified oligonucleotides have T‐bases surrounding the charge neutral backbones. Substitution of T for U is known to slightly increase duplex stability. The 2′OMe/PS control ON1:RNA heteroduplex melted above 75 °C in 200 mM NaCl (Figure S3). To acquire melting temperatures for all backbones and enable comparison among them, melting temperatures against the RNA target were measured at a reduced salt concentration of 25 mM NaCl. The LNA‐amide linkage is moderately stabilising against the RNA target (+4.2 °C) compared to the 2′OMe/PS control, and is very well accommodated within the ON2:DNA duplex, causing a large increase in melting temperature (+7.0 °C). Here, we report the first incorporation of the LNA‐carbamate linkage within an ASO which contains 2′OMe/PS therapeutic modifications. Pleasingly, the duplex stabilisation properties are equivalent to previous reports which incorporate the linkage in an unmodified DNA phosphodiester oligonucleotide; the LNA‐carbamate linkage is only mildly destabilising against both the DNA and RNA targets.^[^ ^17^ ^]^ The LNA‐alkoxyamide linkage is minimally stabilising against both DNA and RNA targets (+1.5 and + 1.7 °C, respectively versus 2′OMe/PS control). The greater destabilising effect of the carbamate relative to the alkoxyamide and amide may be due to the former's increased rigidity, as discussed in the molecular dynamics section. The LNA‐sulfamate is extremely stabilising against both the DNA and RNA targets (+10.5 and + 10.0 °C, respectively compared to the 2′OMe/PS control). LNA alone has been shown to increase duplex stability by up to + 7 °C per modification in unmodified phosphodiester duplexes;^[^ ^18^ ^]^ the closer an analogue is to a phosphodiester, the greater will be the positive influence of the LNA sugar. To evaluate the specific effect of LNA in our 2′OMe/PS sequence we have carried out UV melting on the LNA control oligonucleotides used in this study. The results confirm the extreme stabilisation conferred by LNA and indicate that this is moderated by the charge neutral backbone linkages (Table S2b and Figure S2a,b).

Circular dichroism (CD) was used to evaluate changes in global duplex structure by measurement of polarized light absorption from 200 to 340 nm (Figure 3b). Comparison of ON2‐5:DNA duplexes with ON1:DNA duplexes show that LNA‐charge neutral linkages do not significantly perturb the overall duplex structure, which is consistent with a B‐type helix (Figure S4). However, slight differences can be seen within the CD spectra of the ON:RNA duplexes. Typically, ON:RNA hybrids adopt A‐like conformations rather than B‐like conformations, which are characterised by negative and positive peaks at 210 and 270 nm, respectively.^[^ ^41^ ^]^ The LNA‐amide RNA duplex (ON2:RNA) appears to have a greater maximum toward 260 nm, indicating a larger A‐like character than the ON1 control. The global structure of the LNA‐carbamate and the LNA‐alkoxyamide RNA duplexes display similar features to ON1:RNA (e.g., minima and maxima at 210 and 270 nm, respectively), but the LNA‐alkoxyamide displays a striking increased intensity of ellipticity at 210 nm, indicating additional A‐type structural characteristics. Incorporation of the LNA‐sulfamate linkage (ON5:RNA) induces mixed A/B topology, characterised by a bathochromic shift from 270 nm toward 280 nm and a less intense minimum at 210 nm.^[^ ^42^ ^]^

There is good agreement between the molecular dynamics data and the observed biophysical properties. The most stable linkage (LNA‐sulfamate, ON5) populates the most similar dihedral angles and linker distances to the native phosphate diester, with a seemingly higher degree of flexibility than the other linkers (Figure 2d and S22). This observation is consistent with the increased A→B mixed topology observed in the CD spectrum of ON5:RNA, as the shift from A‐ to B‐form duplexes is accompanied by an increase in global flexibility compared to the native DNA:RNA heteroduplex. Furthermore, this increased flexibility is experimentally confirmed by a faster migration through a TBE‐urea gel (Figure 3e). The observed thermal stability of the LNA‐sulfamate may be in part due to the increased strength of the hydrogen bonding at the 5′‐side thymidine.

Enzymatic stability of modified ONs is important to ensure optimal biological half‐life and therapeutic efficacy. Oligonucleotides were incubated with human serum to determine the endonuclease resistance over 96 h at 37 °C (Figure 3e). An unmodified DNA phosphodiester control was included to verify the activity of the endonucleases. As expected, the DNA control was fully digested, while the 2′OMe/PS control oligonucleotide (ON1) was found to be highly resistant to endonuclease digestion. The addition of LNA‐charge neutral modifications within the PS backbone did not alter the excellent nuclease resistance achieved by the phosphorothioate linkages (see Figure S5 for full gel). It is promising for future in vivo investigations that the LNA‐charge neutral linkages are just as resistant as the standard 2′OMe/PS modifications used in the clinic.

### Splice‐Switching Activity Assay

To evaluate the Splice‐switching Activity of ON1‐5, We Used the HeLa pLuc/705 cell line, which carries a mutated luciferase‐encoding gene. This mutation is an inserted intron which results in incorrect mRNA splicing and the production of non‐functional luciferase protein.^[^ ^21^ ^]^ The oligonucleotides studied are complementary to this aberrant splice site; successful splice‐switching activity by the ON results in functional luciferase production. When treated with luciferin, the magnitude of luminescence is proportional to the quantity of protein produced, and thus, this assay can be used to determine the degree to which the different neutral linkages influence splice‐switching activity.

To compare biological activity independent of cell uptake, oligonucleotides were delivered into the cells using Lipofectamine 2000, a cationic liposome transfection reagent. Following transfection up to 50 nM and gymnosis up to 20 µM, no oligonucleotides in this study displayed significant cytotoxicity (Figure S24). All oligonucleotides were active in the transfection assay (Figure S25A) with the exception of a scrambled sequence control which was used to verify that luciferase production is sequence dependent (Figure S25B). At all doses investigated (12.5 – 100 nM), oligonucleotides containing the LNA‐carbamate (ON3) or LNA‐alkoxyamide (ON4) had comparable splice‐switching activity to the 2′OMe/PS control (ON1). In our previous reports, the addition of four LNA‐amide linkages increases splice‐switching activity following lipofection at low doses (6.25 – 25 nM).^[^ ^16^ ^]^

Here, we show that the addition of two LNA‐amide linkages leads to an increase in activity over ON1 by approximately 2‐fold at all concentrations (12.5– 100 nM). Finally, the addition of the LNA‐sulfamate linkage significantly improved the splice‐switching activity relative to 2′‐OMe/PS ON1 at all concentrations. (Figure S25A).

**Figure 4:**
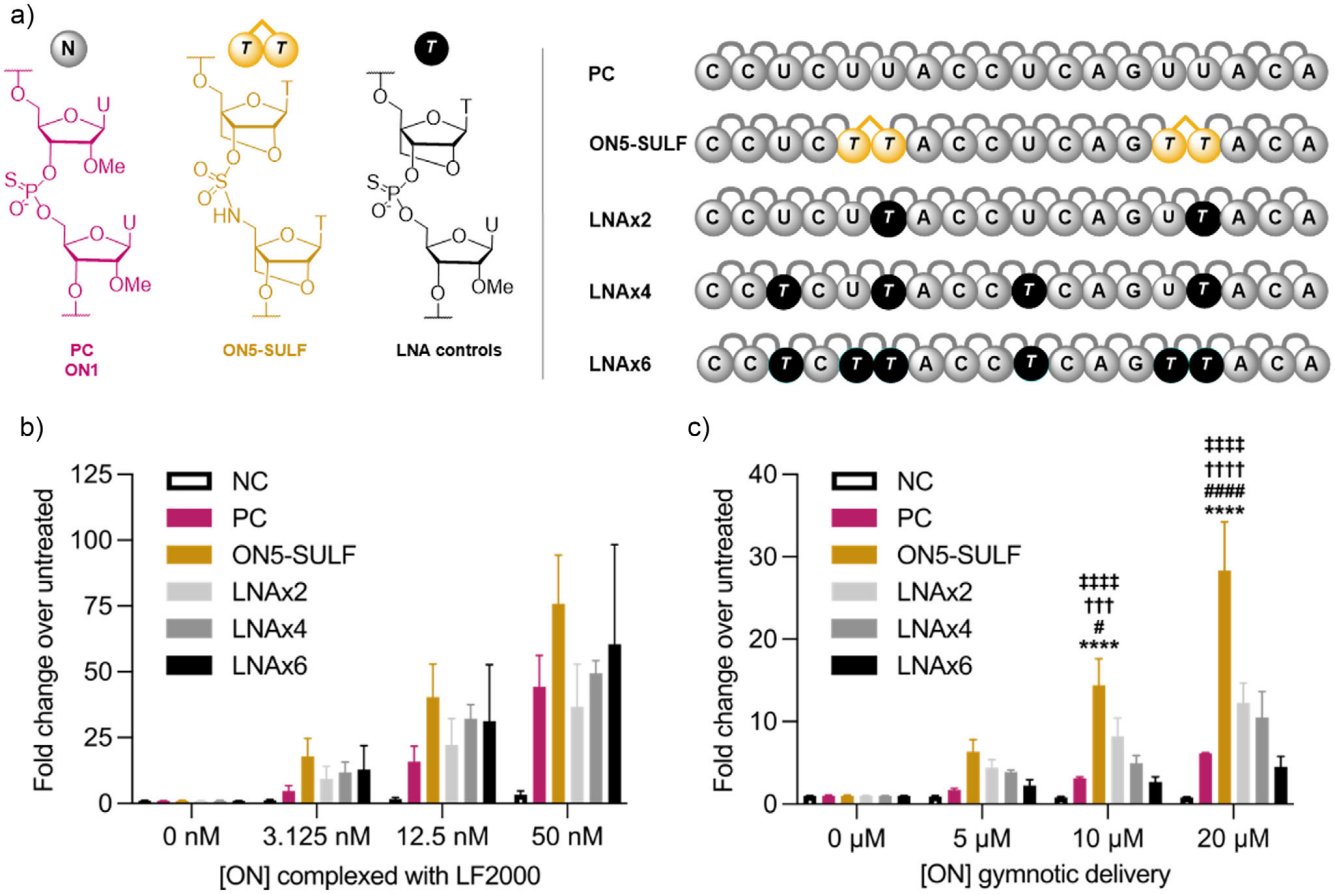
Splice‐switching activities of ONs using a luciferase reporter assay in the HeLa pLuc/705 cell line. Activity was measured as luminescence normalized first to total protein quantity and then to untreated cells (i.e., 0 nM treatment). a) Chemical structures of backbone modifications and oligonucleotide sequences. NC is 2′OMe/PS negative control oligonucleotide UCACUCAGAUAGUUGAAGCC. b) Dose response for ONs transfected into HeLa pLuc/705 cells at the indicated concentrations using Lipofectamine 2000 (LF2000). Luminescence was measured 48 h later. c) Dose response for ONs applied to HeLa pLuc/705 cells at the indicated concentrations in the absence of a transfection reagent. Luminescence was measured 72 h later. Data are means ± SEM for three biological replicates (*n* = 3), where each biological replicate was performed in technical triplicate. Statistics are two‐way analysis of variance (ANOVA) with Dunnett's multiple comparisons test against PC (*), LNAx2 (#), LNAx4 (†), or LNAx6 (‡), *α* = 0.05: */#/†/‡ represents *p* ≤ 0.05, **/##/††/‡‡ represents *p* ≤ 0.01, ***/###/†††/‡‡‡ represents *p* ≤ 0.001, and ****/####/††††/‡‡‡‡ represents *p* ≤ 0.0001.

**Figure 5:**
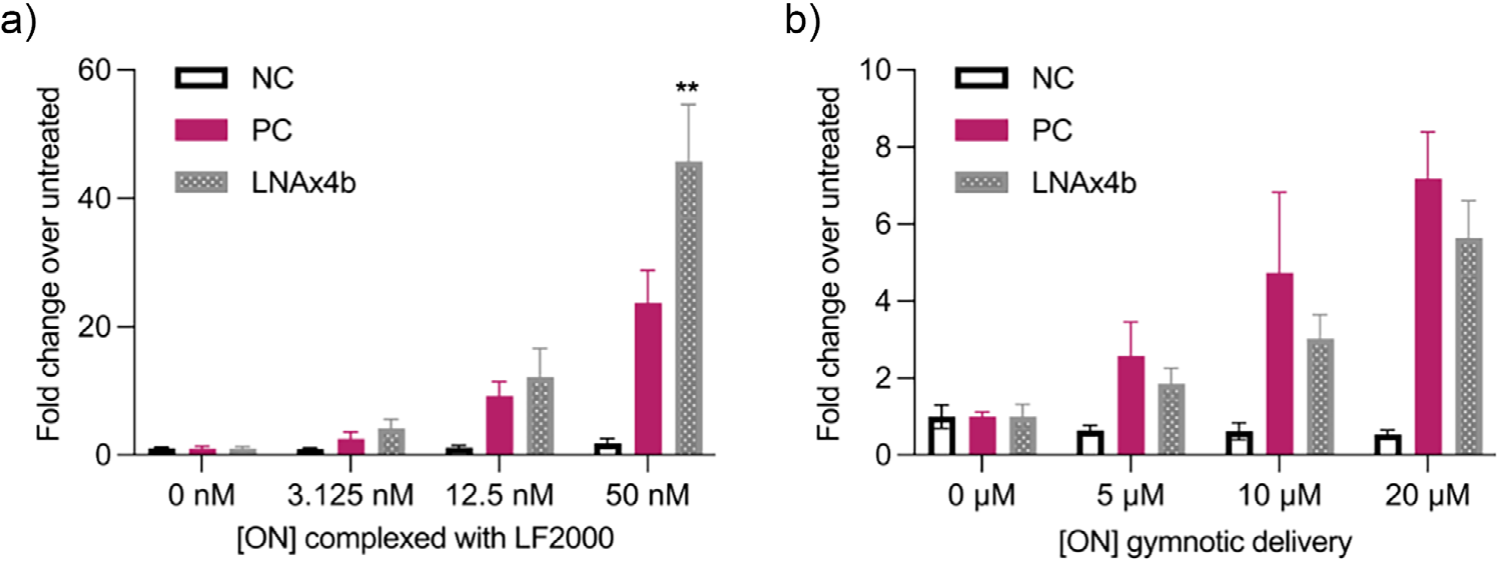
Splice‐switching activity of control oligonucleotide LNAx4b CCUCTTACCUCAGTTACA, where T nucleotides are LNA and there are no charge neutral linkages, compared to PC and NC controls using the luciferase reporter assay in the HeLa pLuc/705 cell line: A. Transfection study and B. Gymnosis study. Conditions are the same as those in Figure 4. Data are means ± SEM for three to five biological replicates (*n* = 3–5), where each biological replicate was performed in technical triplicate. Statistics are two‐way ANOVA with Dunnett's multiple comparisons test against PC, *α* = 0.05: * represents *p* ≤ 0.05, ** represents *p* ≤ 0.01, *** represents *p* ≤ 0.001, and **** represents *p* ≤ 0.0001.

Next, we compared the most active of the above oligonucleotides, LNA‐sulfamate ON5, with the 2′OMe/PS control ON1 and a series of LNA control oligonucleotides (Figure 4). Again, all oligonucleotides except the scrambled negative control were active when transfected, confirming that the sulfamate linkage is compatible with splice correction. Gymnotic conditions are more important for advancing novel chemistries toward therapeutic applications because they more closely represent delivery in vivo, where transfection reagents cannot be used. Pleasingly, the LNA‐sulfamate displayed greatly increased gymnotic activity over both the 2′OMe/PS control ON1 and the controls that contain LNA sugars attached via PS linkages. This shows that the enhanced activity of ON5 is not due to the presence of LNA modifications alone. To confirm this finding, we evaluated the activity of a control oligonucleotide modified with two tandem LNA T phosphorothioate nucleotides as in ON5‐SULF, but without sulfamate linkages (LNAx4b). Relative to the “non‐LNA” 2′OMe/PS control ON1, the LNAx4b control displayed greater activity when transfected (Figure 5a), but decreased activity when delivered via gymnosis (Figure 5b), which is consistent with the trend observed for LNA oligonucleotides with increasing numbers of LNA sugars (Figure 4). Because the gymnotic activity of LNAx4b is lower than that of the non‐LNA positive control, we conclude that LNAx4b is much less active than the LNA‐sulfamate ON5. The observation that the decreased gymnotic activity associated with increased LNA content is negated by the sulfamate linkage is potentially important from a drug development perspective. We do not yet know the origin of the improved activity of LNA‐sulfamate ON5, but it may be related to enhance cellular uptake, better nuclear localization, improved access to the target pre‐mRNA and/or a reduction in non‐productive protein binding in cells. At any rate, the gymnotic activity of ON5 is promising as the LNA‐sulfamate phosphoramidite dimer **17** requires a shorter synthesis than LNA‐amide phosphoramidite dimer **11** which also displays good splice‐switching activity. While they share the synthesis of a locked 5′‐NH_2_ monomer, the LNA‐amide requires a 3′‐COOH monomer, synthesized over 8 steps. In contrast, the LNA‐sulfamate only requires access to the commercially available DMT‐LNA nucleoside **2**. Therefore, the synthesis of all nucleobase combinations of the LNA‐sulfamate dimer will be more synthetically accessible than the LNA‐amide, making it a more practical backbone for further exploration in the oligonucleotide therapeutics field. Because of this we intend to develop an efficient phosphoramidite‐compatible solid‐phase sulfamate monomer coupling strategy to replace the dimer method used in this study.

## Conclusion

In conclusion, we report the novel synthesis of LNA‐amide and LNA‐alkoxyamide dinucleotide phosphoramidites. We incorporated the dimers, in addition to the LNA‐carbamate and LNA‐sulfamate phosphoramidites, into chimeric 2′OMe/PS oligonucleotides via solid‐phase synthesis. For the first time, we have modelled LNA‐charge neutral linkages in molecular dynamics simulations to investigate the implications of these chemical modifications on duplex conformation, specifically flexibility and structural deviation from native heteroduplexes. We have experimentally determined the thermal duplex stability of the LNA‐charge neutral linkage‐containing ONs against DNA and RNA targets and measured their global duplex conformations in solution. Finally, we have investigated therapeutically important properties such as nuclease stability, cytotoxicity, and splice‐switching activity.

In line with previous reports, the LNA‐amide is more active in the splice‐switching luciferase reporter assay than the 2′OMe/PS control (ON1). We show that only two incorporations of the LNA‐amide are sufficient to give enhanced activity (four are reported in the literature).^[^ ^16^ ^]^ Additionally, we show that the LNA‐sulfamate (ON5) backbone has a similar, if not improved, therapeutically relevant properties to LNA‐amide, but with the added advantage of greater synthetic accessibility.

Future work will involve a deeper investigation of the therapeutic properties of oligonucleotides containing varying numbers of LNA‐sulfamate linkages, including cellular trafficking and protein binding profiles. We also propose to study their incorporation into RNase H‐targeting gapmer ASOs and siRNAs. Toxicity has been an obstacle to the clinical use of LNA oligonucleotides.^[^ ^43^, ^44^ ^]^ It will therefore be important to study the biological properties of oligonucleotides containing LNA‐sulfamate linkages in depth to determine if their toxicological and pharmacokinetic properties are more favourable than those of conventional LNA‐phosphodiesters. We are also encouraged to synthesise sulfamate oligonucleotides containing other therapeutically relevant sugars such as 2′‐methoxyethyl and 2′‐fluoro. Finally, oligonucleotides containing sulfamate linkages also have a reduced phosphorothioate content, and this could mediate the known undesirable interactions of phosphorothioates with serum and paraspeckle proteins in vivo.^[^ ^45^ ^]^

## Author Contributions

A.K., L.L., and B.Z.K. carried out small molecule synthesis, supervised by A.H.E.S. and Y.R.B.; A.H.E.S., T.B., and D.D. synthesized the oligonucleotides and A.K., L.L., and D.D., purified them. M.F., supervised by F.D., carried out all computational work. D.D., A.K. and L.L. performed physical and biological experiments and A.H. and A.R. performed biological experiments in the laboratory of M.J.A.W.; L.L. was supervised by T.B., Y.B., and A.H.E.S.; A.K., L.L., M.F., and T.B. wrote the manuscript. All authors contributed to the editing of the manuscript.

## Conflict of Interests

TB and AHES are Inventors on US patent application 63/764 021 filed by Oxford University Innovation on 27/02/2025.

## Supporting information



Supporting Information

## Data Availability

The data that support the findings of this study are available in the Supporting Information of this article.
