# Supplementary material for: Synthesis, Biological Activity, and Molecular Dynamics Simulations of LNA‐Charge Neutral Linkages for Enhanced Splice‐Switching Antisense Oligonucleotides
Source: Angew Chem Int Ed Engl. 2025 Sep 21;64(46):e202511386. doi: 10.1002/anie.202511386 (PMC12603973; doi:10.1002/anie.202511386)
Supplement: Supplementary file 1 — Supporting Information [file ANIE-64-e202511386-s001.docx]

**Supporting Information**

Synthesis, Biological Activity, and Molecular Dynamics Simulations of LNA-Charge Neutral Linkages for Enhanced Splice-Switching Antisense Oligonucleotides

^#^Alice Kennett^[a]^, ^#^Lillian Lie^[a]^, Martin Flerin^[a]^, Belma Zengin Kurt^[a,b]^, Ysobel R. Baker^[a,c]^, Alyssa C. Hill^[d]^, Abinaya Ramesh^[d]^, Matthew J.A. Wood^[d]^, Debashis Dhara^[a]^,Afaf H. El-Sagheer^[a,c] *^, Fernanda Duarte^[a]*^ and Tom Brown^[a]*^

# Equal contributors

* Corresponding authors

[a] Department of Chemistry, University of Oxford, Chemistry Research Laboratory, 12 Mansfield Road, Oxford, OX1 3TA, UK

Emails: fernanda.duartegonzalez@chem.ox.ac.uk, [ahes@soton.ac.uk](mailto:ahes@soton.ac.uk), tom.brown@chem.ox.ac.uk

[b] Department of Pharmaceutical Chemistry, Bezmialem Vakif University, Faculty of Pharmacy, 34093, Istanbul, Türkiye

[c] School of Chemistry, University of Southampton, Highfield, Southampton, SO17 1BJ, UK

[d] Department of Paediatrics, Institute of Developmental and Regenerative Medicine (IDRM), University of Oxford, Oxford OX3 7TY, UK

**Table of Contents**

[List of oligonucleotides 2](#_Toc198890096)

[Alternative sulfur-containing backbones 3](#_Toc198890097)

[Representative UV melting curves 4](#_Toc198890098)

[UV melting curves for ON1 at varying salt concentrations 6](#_Toc198890099)

[Circular dichroism spectra 6](#_Toc198890100)

[Human serum stability study 7](#_Toc198890101)

[Computational methods 8](#_Toc198890102)

[RESP generation 8](#_Toc198890103)

[Topology preparation 9](#_Toc198890104)

[MD simulations 9](#_Toc198890105)

[Analysis and visualisation 9](#_Toc198890106)

[Supplementary results 11](#_Toc198890107)

[RMSD evolution for all simulations 12](#_Toc198890108)

[Full duplex RMSD evolution for all simulations 16](#_Toc198890109)

[Linker distance statistics 22](#_Toc198890110)

[Cell viability assays 23](#_Toc198890111)

[Luciferase assay transfection activity 25](#_Toc198890112)

[Luciferase assay scramble activity 26](#_Toc198890113)

[Experimental 26](#_Toc198890114)

[Oligonucleotide synthesis 26](#_Toc198890115)

[Oligonucleotide characterisation 27](#_Toc198890116)

[Biological assays 27](#_Toc198890117)

[Chemistry 29](#_Toc198890118)

[NMR spectra of compounds 35](#_Toc198890119)

[UPLC and MS analysis of oligonucleotides with LNA-neutral linkages 46](#_Toc198890120)

[References 52](#_Toc198890121)

# List of oligonucleotides

**Table S1.** Oligonucleotides (ONs) used in this study with calculated and found mass spectrometry (MS) data. HPLC-MS traces for all ONs are given in Figure Ss 44-50.

T indicates a locked sugar and * indicates a modified neutral linkage in place of a phosphorothioate. All other sugars are 2’OMe and all other linkages are phosphorothioate.

| **ON** | **Sequence (5’ → 3’)**  (* = modified linkage) | **Expected (Da)** | **Found (Da)** |
| --- | --- | --- | --- |
| *2’OMe/PS on-target splice-switching control oligonucleotide* | | | |
| ON1^PS control^ (**PC**) | CCUCUUACCUCAGUUACA | 6097.9 | 6098.6 |
| *Splice-switching oligonucleotides containing modified linkage* | | | |
| ON2^LNA-amide^ | CCUCT*TACCUCAGT*TACA | 6036.5 | 6036.8 |
| ON3^LNA-carbamate^ | CCUCT*TACCUCAGT*TACA | 6040.4 | 6040.7 |
| ON4^LNA-alkyoxyamide^ | CCUCT*TACCUCAGT*TACA | 6070.1 | 6070.0 |
| ON5^LNA-sulfamate^ | CCUCT*TACCUCAGT*TACA | 6112.4 | 6112.7 |
| *Splice-switching oligonucleotide scrambled control* | | | |
| ON6^PS scramble^ (**NC**) | UCACUCAGAUAGUUGAAGCC | 6935.4 | 6935.5 |
| *Reverse complementary oligonucleotides used in UV melting and CD studies* | | | |
| ON7^DNA_reverse-complement^ | TGTAACTGAGGTAAGAGG | 5627.8 | 5628.0 |
| ON8^RNA_reverse-complement^ | UGUAACUGAGGUAAGAGG | 5859.6 | 5859.5 |

**Table S2a.** Comparison of the melting temperatures (*T*_m_s) of duplexes containing modified neutral linkages flanked by LNA on both sides hybridised to DNA or RNA.

|  | **Sequence (5’ → 3’)** | **DNA**  *T*_m_ *(*∆ *T*_m_) | **RNA**  *T*_m_ *(*∆ *T*_m_) |
| --- | --- | --- | --- |
| ON1^PS control^ | CCUCUUACCUCAGUUACA | 54.7 ±0.2 | 63.3 ±0.1 |
| ON2^LNA-amide^ | CCUCT*TACCUCAGT*TACA | 61.7 ±0.2 (+7.0) | 67.5 ±0.1 (+4.2) |
| ON3^LNA-carbamate^ | CCUCT*TACCUCAGT*TACA | 53.5 ±0.2 (−1.2) | 61.3 ±0.1 (−2.0) |
| ON4^LNA-alkyoxyamide^ | CCUCT*TACCUCAGT*TACA | 56.2 ±0.1 (+1.5) | 65.0 ±0.1 (+1.7) |
| ON5^LNA-sulfamate^ | CCUCT*TACCUCAGT*TACA | 65.2 ±0.1 (+10.5) | 73.3 ±0.1 (+10.0) |

*T*_m_ values were measured using 3.0 µM concentrations of each oligonucleotide strand in 10 mM phosphate buffer (pH 7.0) containing either 200 mM NaCl for DNA:ASO or 25 mM NaCl for RNA:ASO duplexes, where ASO is the modified oligonucleotide. T indicates a locked sugar and * indicates a modified neutral linkage in place of a phosphorothioate. ∆*T*_m_ = *T*_m_ modified – *T*_m_ control. Representative melting curves are given in Figures S1a and S1b. DNA target = TGTAACTGAGGTAAGAGG; RNA target = UGUAACUGAGGUAAGAGG.

**Table S2b.** Comparison of the melting temperatures (*T*_m_s) of duplexes containing control oligonucleotides containing LNA hybridised to DNA or RNA.

|  | **Sequence (5’ → 3’)** | **DNA**  *T*_m_ *(*∆ *T*_m_) | **RNA**  *T*_m_ *(*∆ *T*_m_) |
| --- | --- | --- | --- |
| ON1^PS control^ | CCU CUU ACC UCA GUU ACA | 49.3 | 54.6 |
| LNA×2 | CCU CUT ACC UCA GUT ACA | 58.6 (+9.7) | 62.7 (+8.1) |
| LNA×4 | CCT CUT ACC TCA GUT ACA | 66.8 (+17.5) | 69.5 (+14.9) |
| LNA×4b | CCU CTT ACC UCA GTT ACA | 64.4 (+15.1)  69.2 (+14.5)* | 67.9 (+13.6)  74.5 (+11.2)* |
| LNA×6 | CCT CTT ACC TCA GTT ACA | 73.1 (+23.8) | Nd |

*T*_m_ values were measured using 2.0 µM concentrations of each oligonucleotide strand in 10 mM phosphate buffer (pH 7.0) containing either 100 mM NaCl for DNA:ASO or no NaCl for RNA:ASO duplexes, where ASO is the LNA control oligonucleotide (**T*_m_ values were measured at 200 mM NaCl for DNA target and 25 mM NaCl for RNA target). T indicates a locked sugar. ∆*T*_m_ = *T*_m_ LNA control oligo – *T*_m_ of 2’-OMe phosphorothioate control (error ±0.25 ̊C). Representative melting curves are given in Figures S2a and S2b. DNA target = TGTAACTGAGGTAAGAGG; RNA target = UGUAACUGAGGUAAGAGG.

# Alternative sulfur-containing backbones

**Scheme S1.** Sulfur-containing linkages considered for investigation as neutral backbones. Only the LNA-sulfamate was investigated in a chimeric splice-switching ASO, because the conversion of amide to thioamide was unsuccessful, the thiocarbamate linkage was incompatible with ammonia deprotection, and the sulfonamide synthesis was lengthy and did not satisfy our desire to find an easily accessible backbone modification.

# Representative UV melting curves


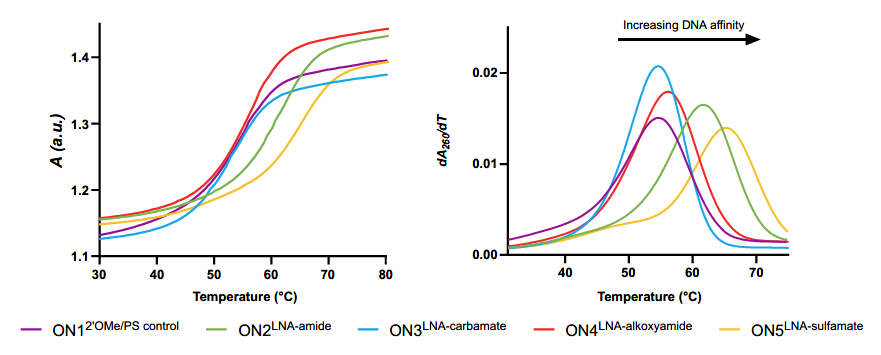


**Figure S1a.** UV melting studies for modified ASOs ON1-ON5 against complementary DNA (ON7) (Table S1). Left. Representative UV melting curves measured using 3 µM of each ON in pH 7.0, 10 mM phosphate buffer containing 200 mM NaCl; Right. 1st derivative of melting curves. The curves shown are representative of three independent repeats, each consisting of three technical repeats. DNA target: 5'-TGTAACTGAGGTAAGAGG-3'


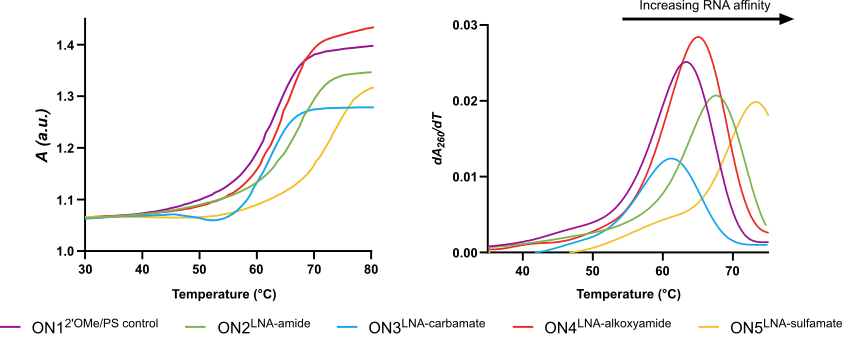

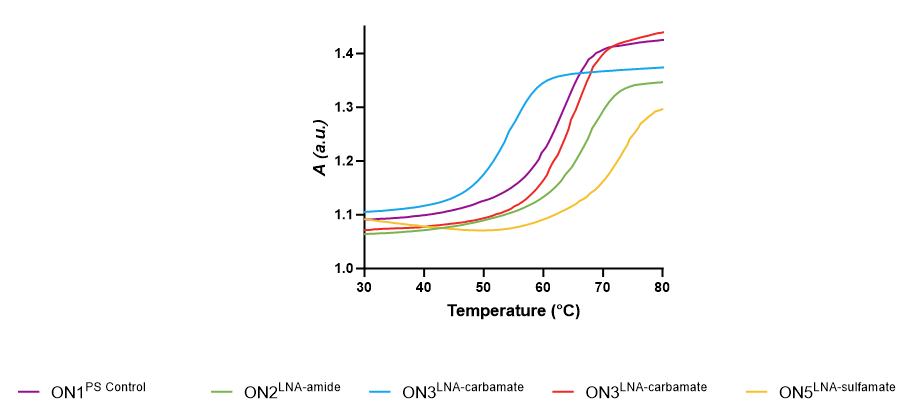


**Figure S1b.** UV melting studies for modified ASOs ON1-ON5 against complementary RNA (ON8) (Table S1). Left. Representative UV melting curves measured using 3 µM of each ON in pH 7.0, 10 mM phosphate buffer containing 25 mM NaCl; Right. 1st derivative of melting curves. The curves shown are representative of three independent repeats, each consisting of three technical repeats. RNA target: 5'-UGUAACUGAGGUAAGAGG-3'


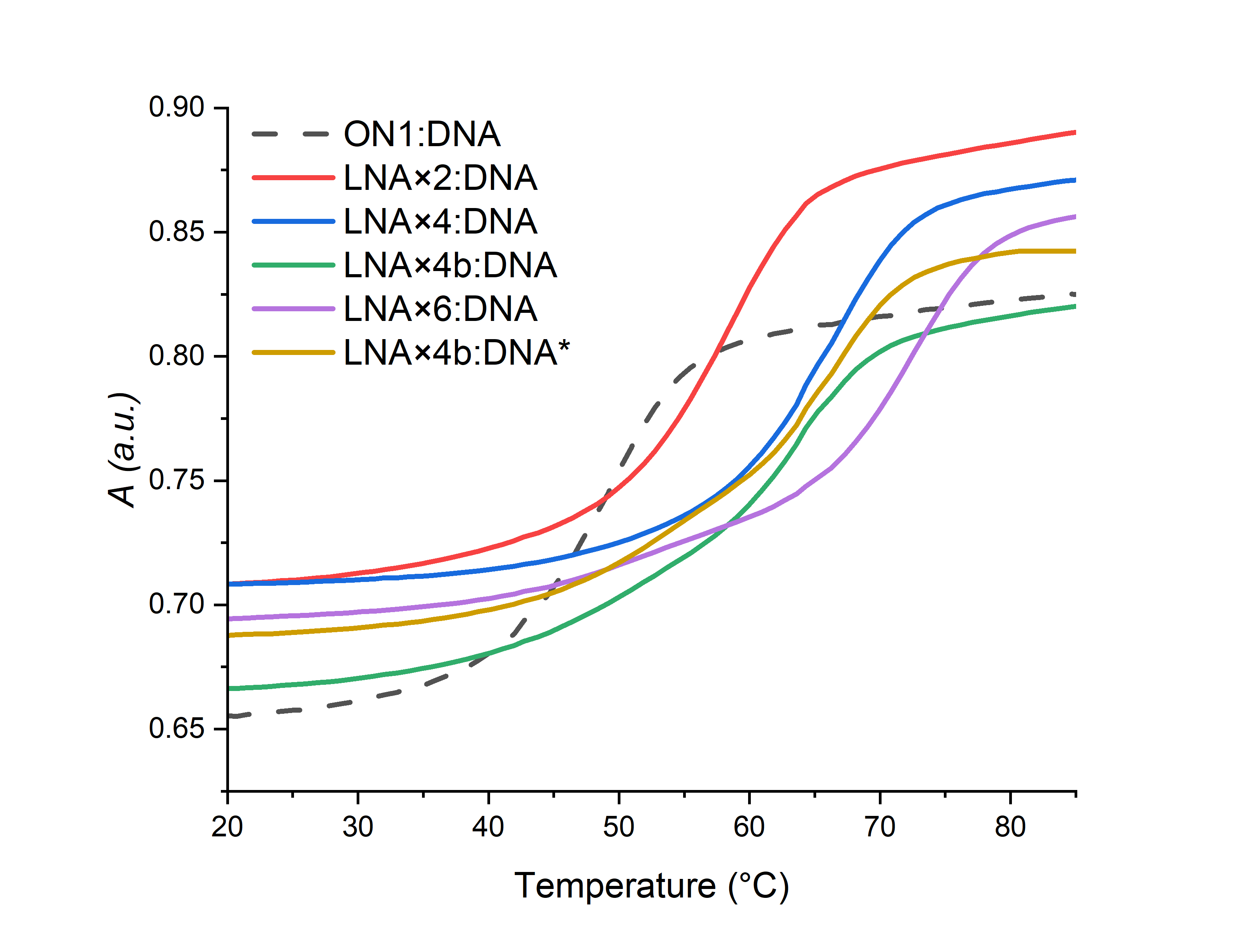

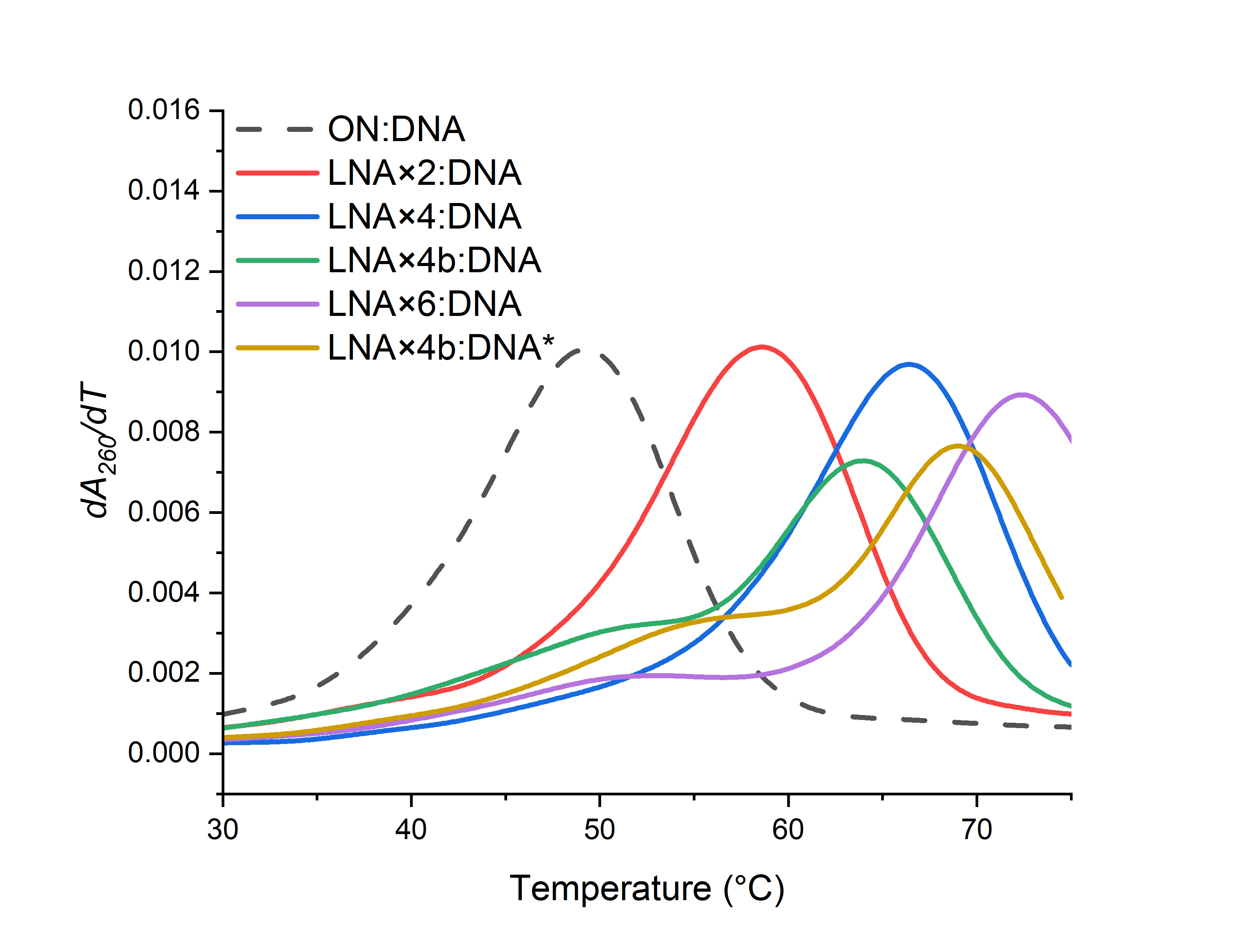


**Figure S2a.** UV melting studies for control LNA oligonucleotides against complementary DNA (ON7) (Table S1). Left. Representative UV melting curves measured using 2 µM of each ON in pH 7.0, 10 mM phosphate buffer containing 100 mM NaCl (*200 mM NaCl); Right. 1st derivative of melting curves. The curves shown are representative of three independent repeats, each consisting of three technical repeats. DNA target: 5'-TGTAACTGAGGTAAGAGG-3'


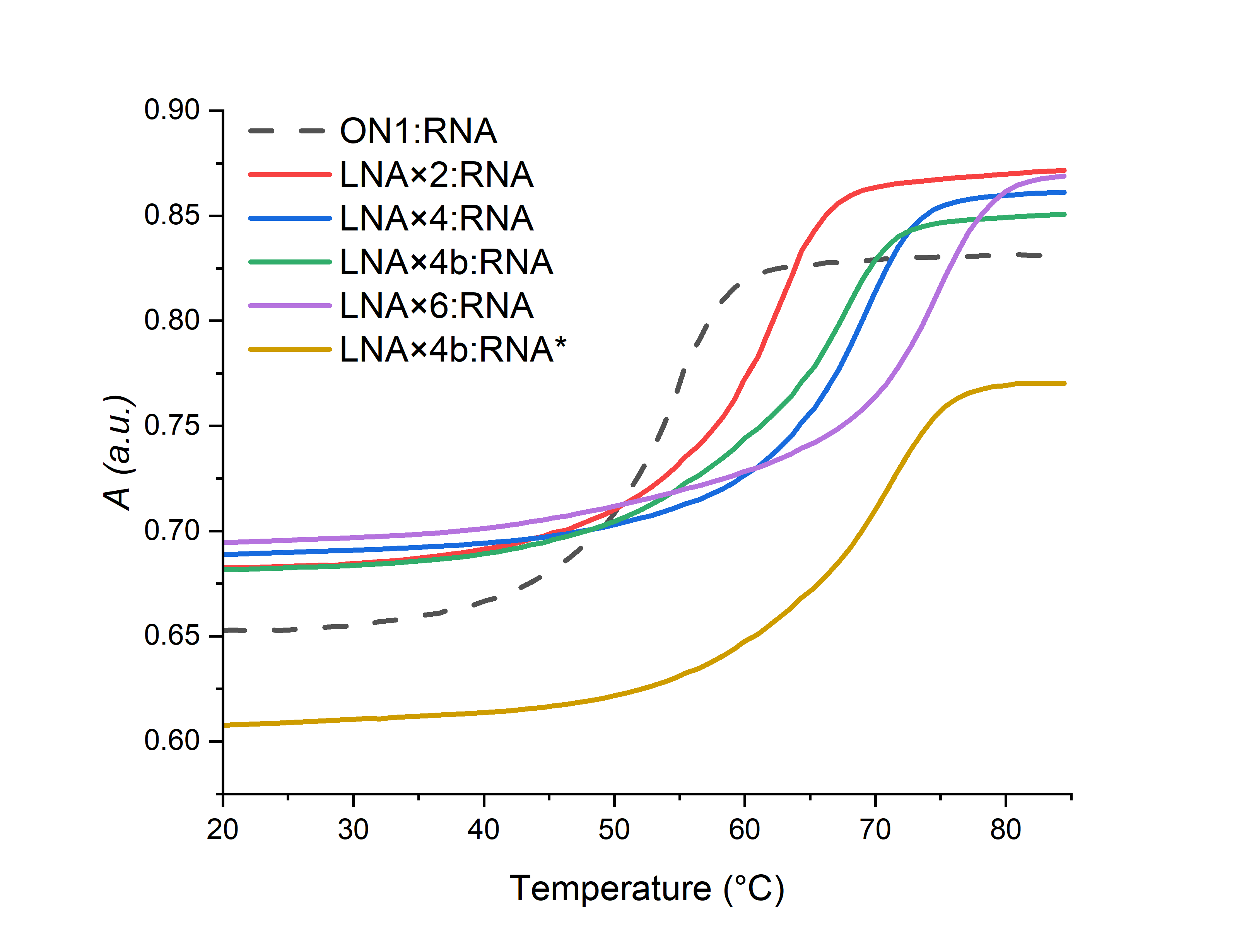

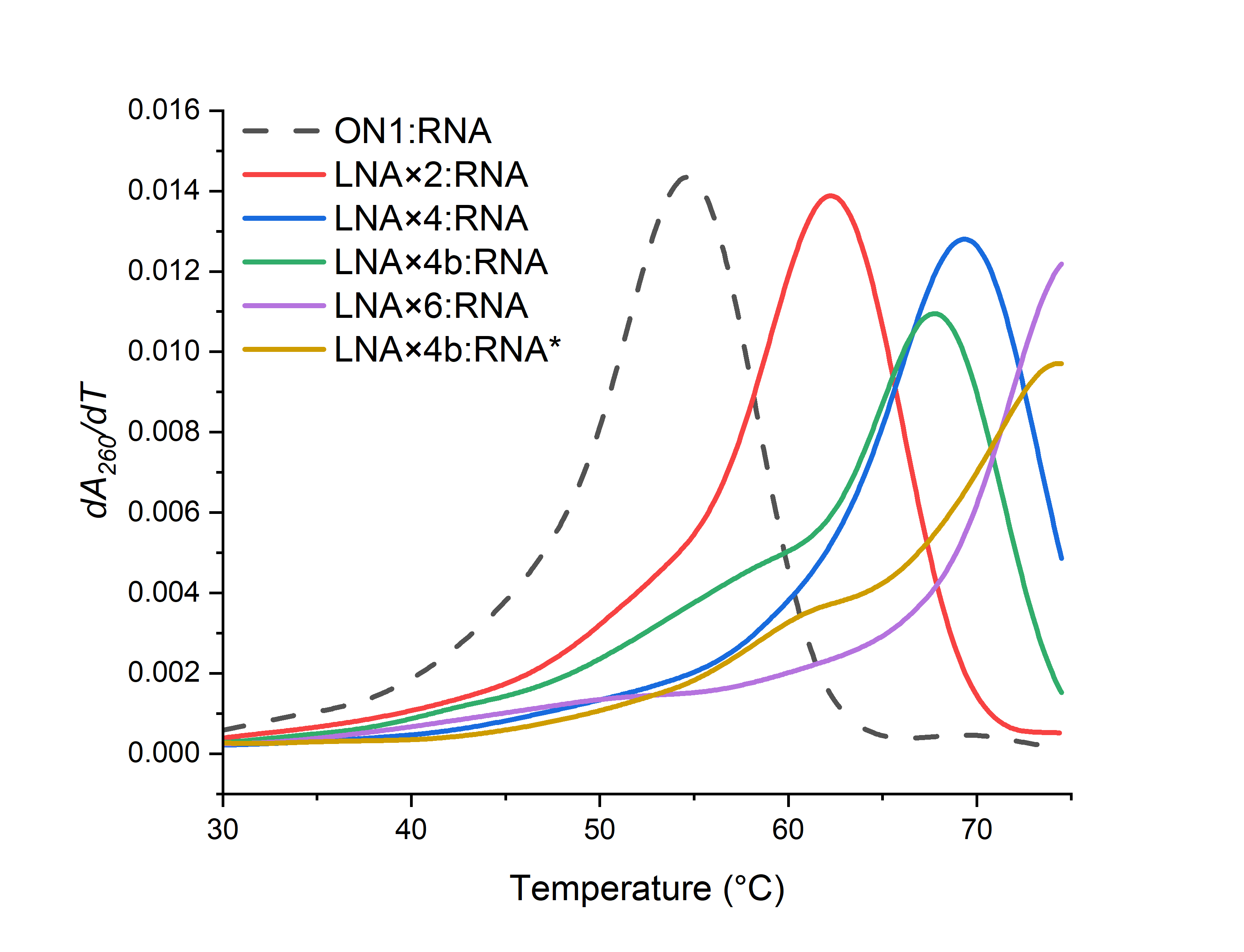


**Figure S2b.** UV melting studies for control LNA oligonucleotides against complementary RNA (ON8) (Table S1). Left. Representative UV melting curves measured using 2 µM of each ON in pH 7.0, 10 mM phosphate buffer containing no NaCl (*25 mM NaCl); Right. 1st derivative of melting curves. The curves shown are representative of three independent repeats, each consisting of three technical repeats. RNA target: 5'-UGUAACUGAGGUAAGAGG-3'

# UV melting curves for ON1 at varying salt concentrations


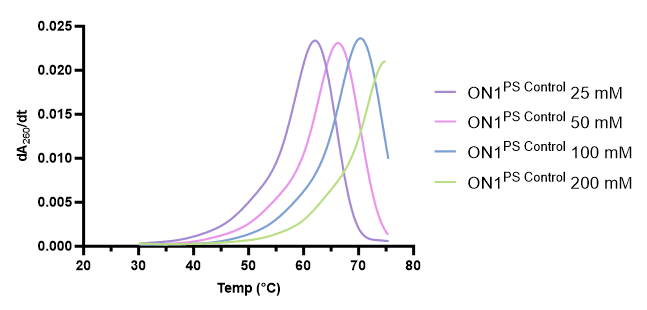


**Figure S3.** Representative UV melting curves measured using 3 µM of ON1 in pH 7.0, 10 mM phosphate buffer with varying concentrations of NaCl. The curves shown are representative of three independent repeats, each consisting of three technical repeats. RNA target: 5'-UGUAACUGAGGUAAGAGG-3'

# Circular dichroism spectra

**Figure S4.** Circular dichroism (CD) spectra of ASOs ON1–ON5 in duplex with a DNA target containing 3 μM of ASO and 3 μM of the DNA target in 10 mM phosphate buffer, 100 mM NaCl, pH 7.0. Data points were taken as an average of three scans at 25 °C. DNA target: 5'-TGTAACTGAGGTAAGAGG-3'

# Human serum stability study


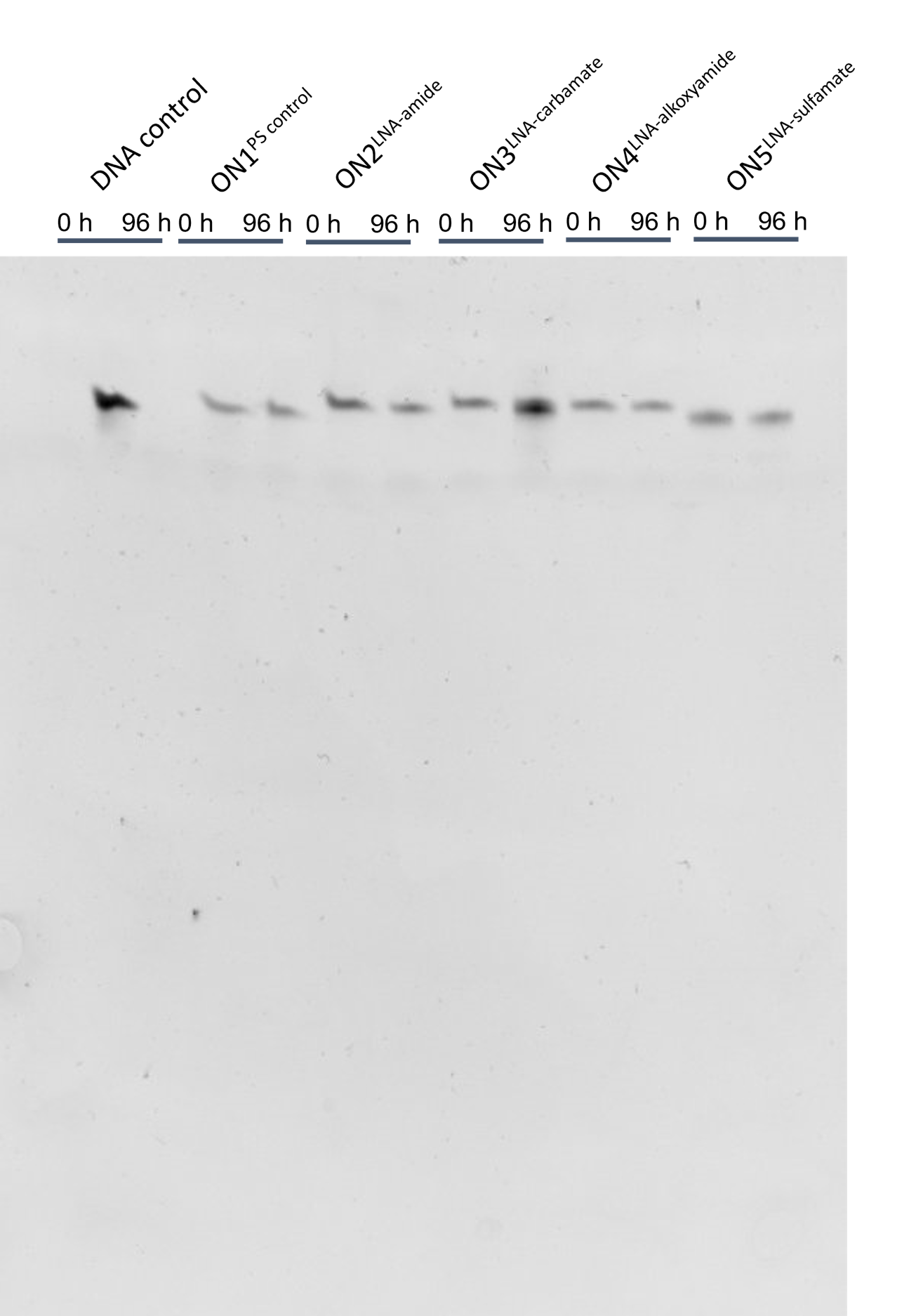


**Figure S5.** Full gel of nuclease resistance of ON1-5 and a DNA phosphate control oligonucleotide against human serum (96 h, 37 °C) (1:1 v/v).

# Computational methods

## RESP generation


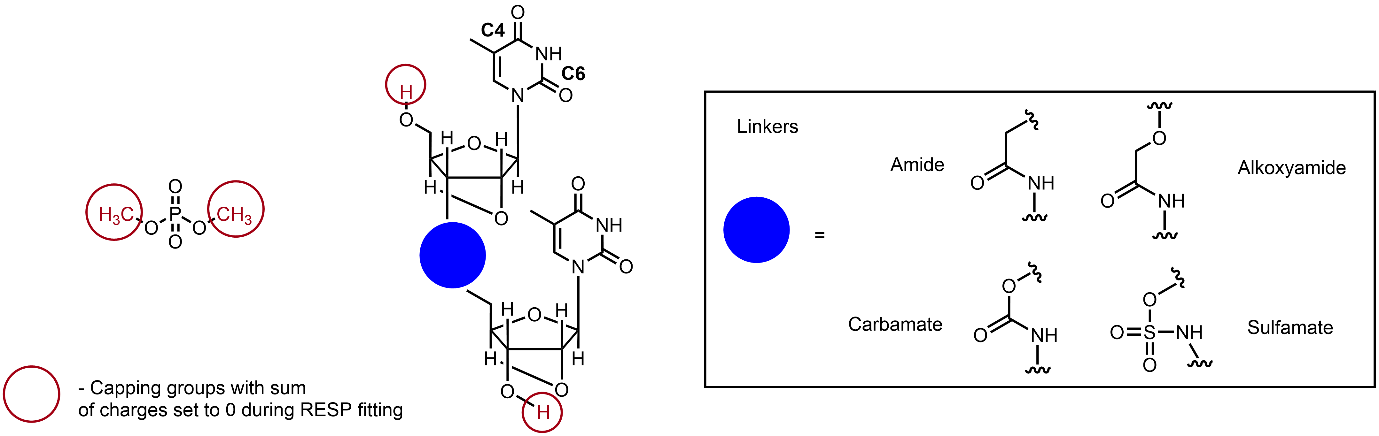


**Figure S6.** The methodology for RESP fitting of the locked thymidine dinucleotides as a single residue adapted from Cieplak et al.[^1^](#_ENREF_1)

RESP fitting was conducted for all systems except the native DNA-RNA heteroduplex in the standard sequence of a geometry optimisation followed by an electrostatic calculation whose density output is finally fitted by the RESP algorithm to produce atomic charges.[^1^](#_ENREF_1),[^2^](#_ENREF_2) Geometry optimisations were performed with ORCA (version 5.0.3) at the ɷ-B97X-D3/def2-SVP level of theory.[^3-6^](#_ENREF_3) The LNA-PO system’s locked thymidines were treated according to the procedure by Cieplak et al, which was also employed in the work of Condon et al optimising the parameters for locked nucleotides, and we found overall good agreement with their RESP charges.[^7^](#_ENREF_7),[^8^](#_ENREF_8) In this protocol, the locked phosphonucleotide is split into the phosphate group and a locked thymidine, with methyl capping groups on the phosphate, and hydrogen capping groups on the 3’ and 5’ oxygens of the sugar of the thymidine. In order to allow for this same protocol to be followed for the different thymidine dinucleotides with neutral linkers, the thymidines flanking these modified linkers were treated as one residue (see Figure S6). This is because the splitting employed by Cieplak, where all internal nucleotides of the same nucleobase are treated equally cannot be carried out across the thymidines surrounding the neutral linkers, as the thymidine on the 5’ of the linker may differ in its charge distribution to that on the 3’ of the linker, due to the unsymmetrical nature of the linkers between them (e.g. an amide is unsymmetric about the carbonyl, whereas a phosphate is symmetrical about the phosphorous). Due to the greater flexibility of a dinucleotide compared to a single nucleotide, it was deemed that restraining the thymine portions of the locked dinucleotides in the optimisation would guarantee that the subsequent electrostatic potential would be performed on a conformation similar to that in the duplex. The thymidine dinucleotides were therefore optimised using distance restraints between the C2 and C4 carbons of each thymidine of 4.7 and 4.1 Å.

Electrostatic potentials were calculated on the optimised geometries in Gaussian 09 at the HF/6-31G* level of theory, using the Merz-Singh-Kollman scheme for charge calculation with 6 density points in each layer (IOp(6/33=2, 6/42=6, 6/50=1)).[^7^](#_ENREF_7) RESP fitting was employed in antechamber, specifying the sum of the charges of the capping groups to be 0.[^9^](#_ENREF_9)

To account for the unique behaviour of the locked nucleotides, the AMBER parm99 parameters for locked uridines by Condon et al were adapted for the locked thymidines used in this study, made possible by the fact that their additional parameters only concern the dihedrals and angles associated with C2 of the uracil ring, which was here assumed to behave similarly to the C2 of thymine.[^8^](#_ENREF_8) Linker atoms were assigned GAFF atom types and their parameters generated in antechamber.[^9^](#_ENREF_9),[^10^](#_ENREF_10) All parameters were converted to the GROMACS format using ParmEd and added to the parm99 forcefield alongside the parameters from Condon et al.[^7^](#_ENREF_7),[^11^](#_ENREF_11)

## Topology preparation

The starting conformations for all simulations containing locked nucleotides were obtained from the crystal structure (PDB entry 7NRP) of the unmodified double helix (d-CTTTTCTTTG/rCAAAGAAAAG).[^12^](#_ENREF_12) For the LNA-PO system, the ribose was modified manually to link the C2’ and C4’ carbons at the underlined nucleotides in the sequence of the DNA oligomer. This structure was then additionally modified and fitted with linkers as optimised in the DFT calculations. The prerequisite for the starting structures of the systems with modified neutral linkers was for the carbonyl bond (or one of the sulfur oxygen double bonds in the sulfamate) to be pointing towards the middle of the duplex rather than away from it, as has been observed in crystal structures of amide linkers between locked nucleotides previously.

## MD simulations

MD simulations were performed in the GROMACS simulation software package (v.2021.3 in all subsequent mentions), employing our modified AMBER parm99 force field.[^7^](#_ENREF_7),[^13^](#_ENREF_13) Water molecules were described by TIP3P water model.[^14^](#_ENREF_14) Default AMBER parm99 sodium ion parameters were used. An example of the .mdp files for each step of the simulations is supplied in the supplementary data.

For all simulations, the double helix was inserted in a cubic box, with a 10 Å buffer between the helix and the box walls, and then solvated with water. The charges were neutralised with the addition of sodium ions. The minimisation-annealing-equilibration sequence followed the protocol outlined by Galindo-Murillo et al.[^15^](#_ENREF_15) First, the system was then minimised using 10000 steps of the steepest descent algorithm, with an initial 10000 kJ mol^-1^ nm^-1^ restraint on all heavy atoms of the duplex. This was followed by simulated annealing from 0 to 300 K over 500 ps while keeping a 10000 kJ mol^-1^ nm^-1^ restraint on heavy atoms. Six rounds of NPT equilibration followed, with 10000, 5000, 3000, 1000, 500 and finally 0 kJ mol^-1^ nm^-1^ restraints on heavy atoms, lasting 500 ps each. Finally, the system was simulated in the NPT ensemble for 1 µs. This was repeated three times for each different system, with random velocities used in the simulated annealing step. The simulations were performed with three-dimensional periodic boundary conditions. Long-range electrostatics were described with the Particle Mesh Ewald (PME) algorithm.[^16^](#_ENREF_16) The temperature of the system was maintained at 300 K using the V-rescale thermostat and a coupling constant of 0.1 ps.[^17^](#_ENREF_17) Pressure was controlled by the C-rescale barostat at 1.0 bar, with an isothermal compressibility of 4.5 × 10-5 bar^–1^.[^18^](#_ENREF_18) All bond lengths involving hydrogen atoms were constrained using the LINCS algorithm.[^19^](#_ENREF_19) Coordinates were saved every 100 ps, and the trajectories were concatenated for subsequent analysis when necessary.

## Analysis and visualisation

The RMSD was calculated using the rms utility of GROMACS by only considering the heavy atoms of the inner six base pairs of the double helix of each system, using these same sets of atoms of the minimised structures (i.e. the structures before any simulated annealing or equilibration) as the reference for fitting. In the comparison plot (Figure 2B), they were plotted as gaussian density kernels – for comparison, the density normalised histograms of RMSD with 100 bins are also shown for the native duplex system. Distances of the linkers were calculated for all frames of each simulation as the distances between the 3’ and 5’ carbons of the relevant neighbouring thymidines for all systems using distance utility of GROMACS and concatenated for the two runs to produce the violin plots.

Clustering was carried out using UCSF Chimera (v.1.17.3 in all further mentions), using every 34^th^ frame of the combined simulation trajectories and was based on the whole duplex structure.[^20^](#_ENREF_20) These structures were then aligned using the MatchMaker utility in Chimera and visualised using the different depictions. The visualisations in fig. 2A depict the structures of each non-native duplex overlayed with the native duplex for ease of comparison of the average clusters. The inserts of fig. 2A showing the linkers were produced using PyMOL (v.3.0.1).[^21^](#_ENREF_21)

Average dihedral angles during the simulation runs were calculated using the GROMACS gangle utility, with the oxygen of the carbonyl bond serving as the origin of the dihedral angle for both dihedral angles (i.e. for the amide the dihedral towards the nitrogen atom was calculated as the angle of O=C-N-C, etc.).

Hydrogen bonding analysis for the simulations was carried out using the nastruct utility of the CPPTRAJ trajectory analysis software (v.4.14.0) as a part of the AmberTools (v.19.12) suite of programs.[^22^](#_ENREF_22),[^23^](#_ENREF_23) The modified thymine residues were mapped onto regular thymidines for analysis, and only the 6 inner base pairs were considered for hydrogen bonding. The percent hydrogen bonding was then calculated for each system as:

$${\%}_{HB}=\frac{\sum_{\text{n=1}}^{\text{n. frames}} \text{n}_{\text{HB}}}{\sum_{\text{n=1}}^{\text{n. frames}} \text{n}_{\text{HB max}}}*100$$

Where n_HB_ is the number of hydrogen bonds present in a frame, with the distance cutoff set as the default 3.5 Å, and n_HB max_ is the maximum number of hydrogen bonds possible in the base pair under consideration (2 for T-A, 3 for G-C). The average hydrogen bonding percentage of the unmodified native control heteroduplex was then subtracted to a give a relative ${\%}_{HB}$ difference for each modified system.

## Supplementary results

Average Structures of Most Populated Clusters


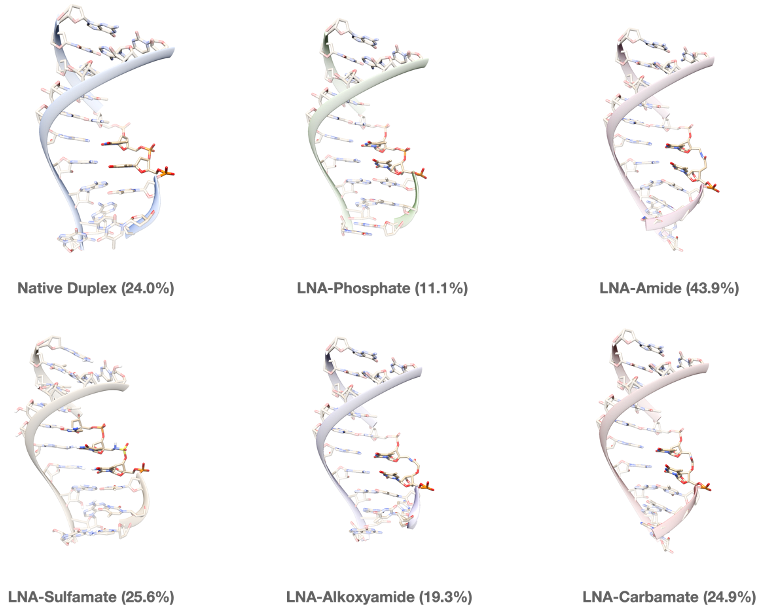


**Figure S7.** Alternative depiction of the averaged most populated clusters for all systems, showing all atoms, with the key thymidine linkers highlighted, and the percentage population of the most occupied cluster shown in brackets.

## RMSD evolution for all simulations

|  |  |
| --- | --- |
|  | |

**Figure S8**. RMSD evolution of the inner 6 base pairs of the DNA-RNA heteroduplex over the simulation time of 1 µs for the native system over three simulation runs, with the rolling average over 100 frames overlaid.

|  |  |
| --- | --- |
|  | |

**Figure S9.** RMSD evolution of the inner 6 base pairs of the DNA-RNA heteroduplex over the simulation time of 1 µs for the LNA-PO linker system over three simulation runs, with the rolling average over 100 frames overlaid.

|  |  | | |
| --- | --- | --- | --- |
|  | | |
|  |  |

**Figure S10.** RMSD evolution of the inner 6 base pairs of the DNA-RNA heteroduplex over the simulation time of 1 µs for the LNA-amide neutral linker system over three simulation runs, with the rolling average over 100 frames overlaid.

|  |  |
| --- | --- |
|  | |  |
|  |  |

**Figure S11.** RMSD evolution of the inner 6 base pairs of the DNA-RNA heteroduplex over the simulation time of 1 µs for the LNA-sulfamate neutral linker system over three simulation runs, with the rolling average over 100 frames overlaid.

|  |  | | |
| --- | --- | --- | --- |
|  | | |
|  |  |

**Figure S12.** RMSD evolution of the inner 6 base pairs of the DNA-RNA heteroduplex over the simulation time of 1 µs for the LNA-alkoxyamide neutral linker system over three simulation runs, with the rolling average over 100 frames overlaid.

|  |  | | |
| --- | --- | --- | --- |
|  | | |
|  |  |

**Figure S13.** RMSD evolution of the inner 6 base pairs of the DNA-RNA heteroduplex over the simulation time of 1 µs for the LNA-carbamate neutral linker system over three simulation runs, with the rolling average over 100 frames overlaid.

## Full duplex RMSD evolution for all simulations

As discussed in the main text, terminal base pair fraying events are common, and result in the RMSD of the entire chain being shifted between 2 and 3 Å relative to the inner 6 base pairs only in a uniform manner.

|  |  |
| --- | --- |
|  | |

**Figure S14**. RMSD evolution entire DNA-RNA heteroduplex over the simulation time of 1 µs for the native system over three simulation runs, with the rolling average over 100 frames overlaid.

|  |  |
| --- | --- |
|  | |

**Figure S15.** RMSD evolution entire DNA-RNA heteroduplex over the simulation time of 1 µs for the LNA-PO linker system over three simulation runs, with the rolling average over 100 frames overlaid.

|  |  | | |
| --- | --- | --- | --- |
|  | | |
|  |  |

**Figure S16.** RMSD evolution entire DNA-RNA heteroduplex over the simulation time of 1 µs for the LNA-amide neutral linker system over three simulation runs, with the rolling average over 100 frames overlaid.

|  |  |
| --- | --- |
|  | |  |
|  |  |

**Figure S17.** RMSD evolution entire DNA-RNA heteroduplex over the simulation time of 1 µs for the LNA-sulfamate neutral linker system over three simulation runs, with the rolling average over 100 frames overlaid.

|  |  | | |
| --- | --- | --- | --- |
|  | | |
|  |  |

**Figure S18.** RMSD evolution entire DNA-RNA heteroduplex over the simulation time of 1 µs for the LNA-alkoxyamide neutral linker system over three simulation runs, with the rolling average over 100 frames overlaid.

|  |  | | |
| --- | --- | --- | --- |
|  | | |
|  |  |

**Figure S19.** RMSD evolution entire DNA-RNA heteroduplex over the simulation time of 1 µs for the LNA-carbamate neutral linker system over three simulation runs with the rolling average over 100 frames overlaid.

Analysis of Significant Jumps in RMSD

| 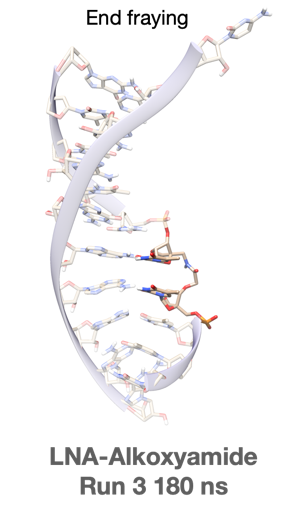 | 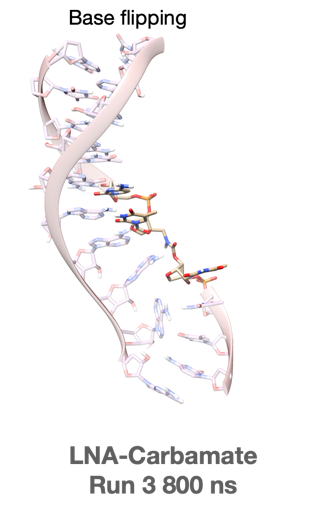 | | | |
| --- | --- | --- | --- | --- |
| 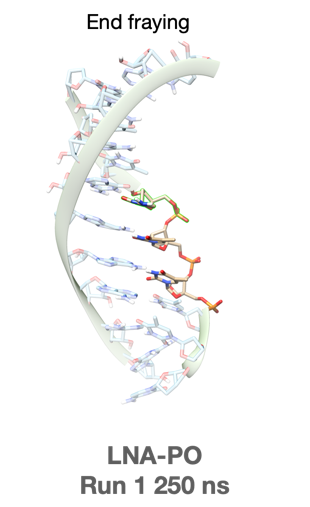 | | 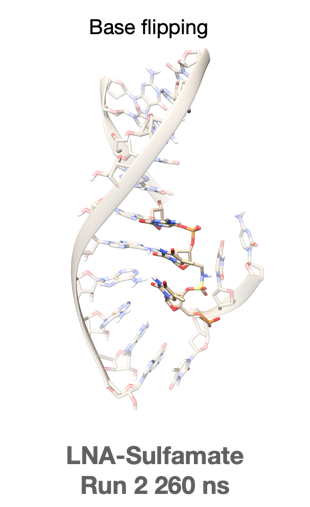 | |
|  |  | |

**Figure S20.** Analysis of the structures at significant RMSD jumps observed in the simulation trajectories, indicating the system and time of the event (below), and the type of event (above).

Linker Dihedral Angle Statistics

The distributions were modelled as gaussians, and the values reported in the main text indicate the middle of the gaussian distribution of each significantly populated dihedral angle distribution peak (more than 20% of the total angle population).


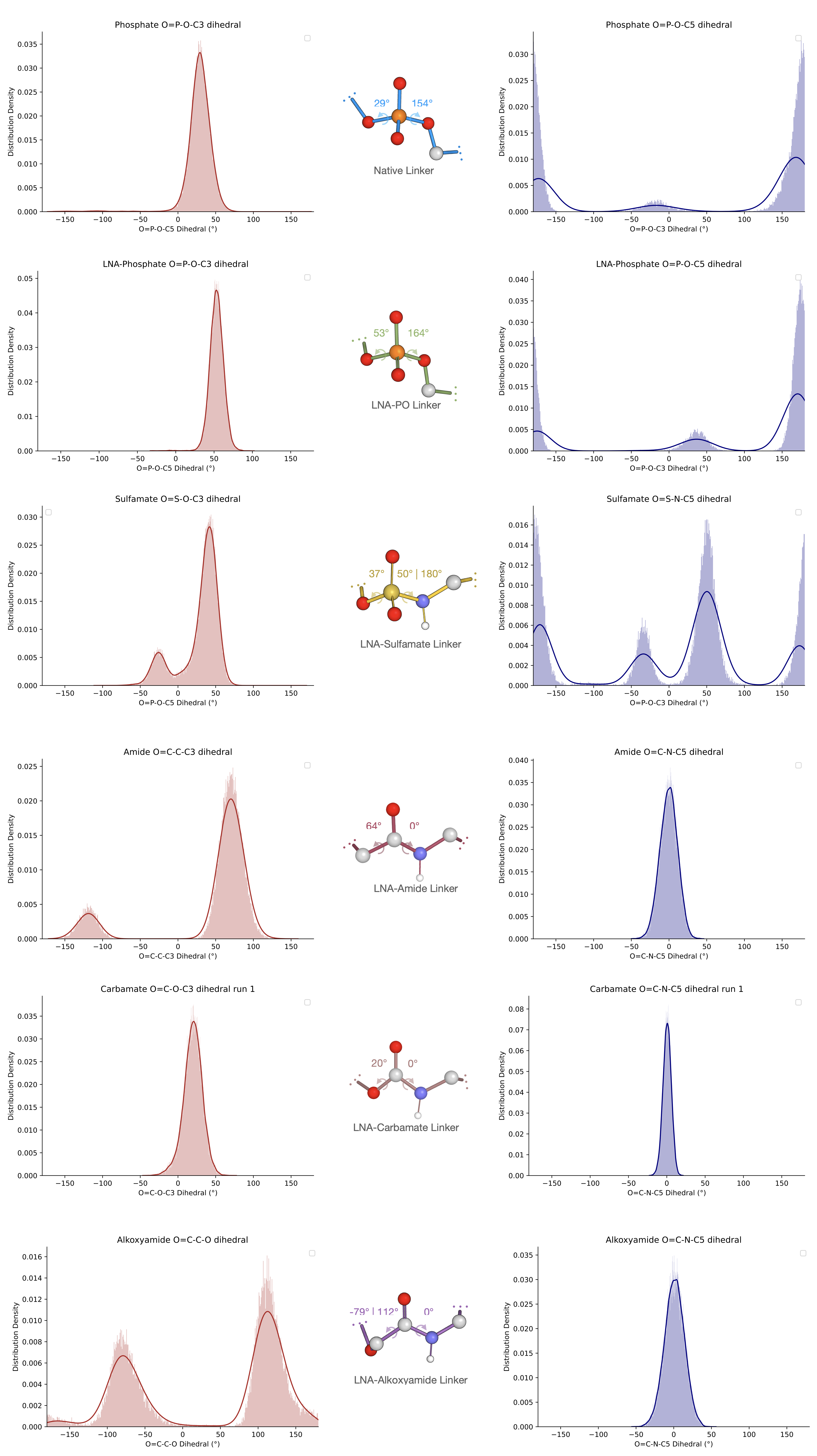


**Figure S21.** RMSD evolution entire DNA-RNA heteroduplex over the simulation time of 1 µs for the neutral linker systems over three simulation runs.

##

## Linker distance statistics


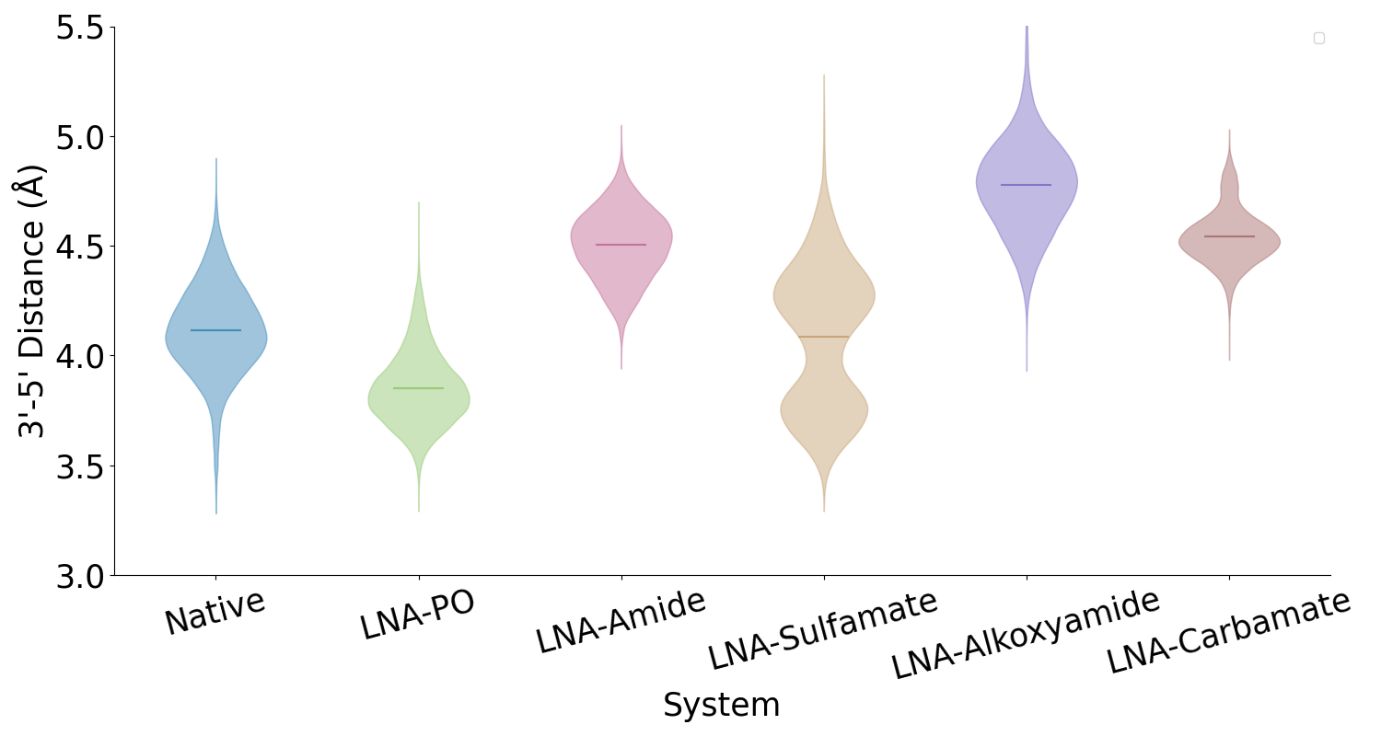


**Figure S22.** Violin plot illustrating the 3’-5’ distance distributions between the two thymidine residues with varying linkers, horizontal line indicates the average distance.


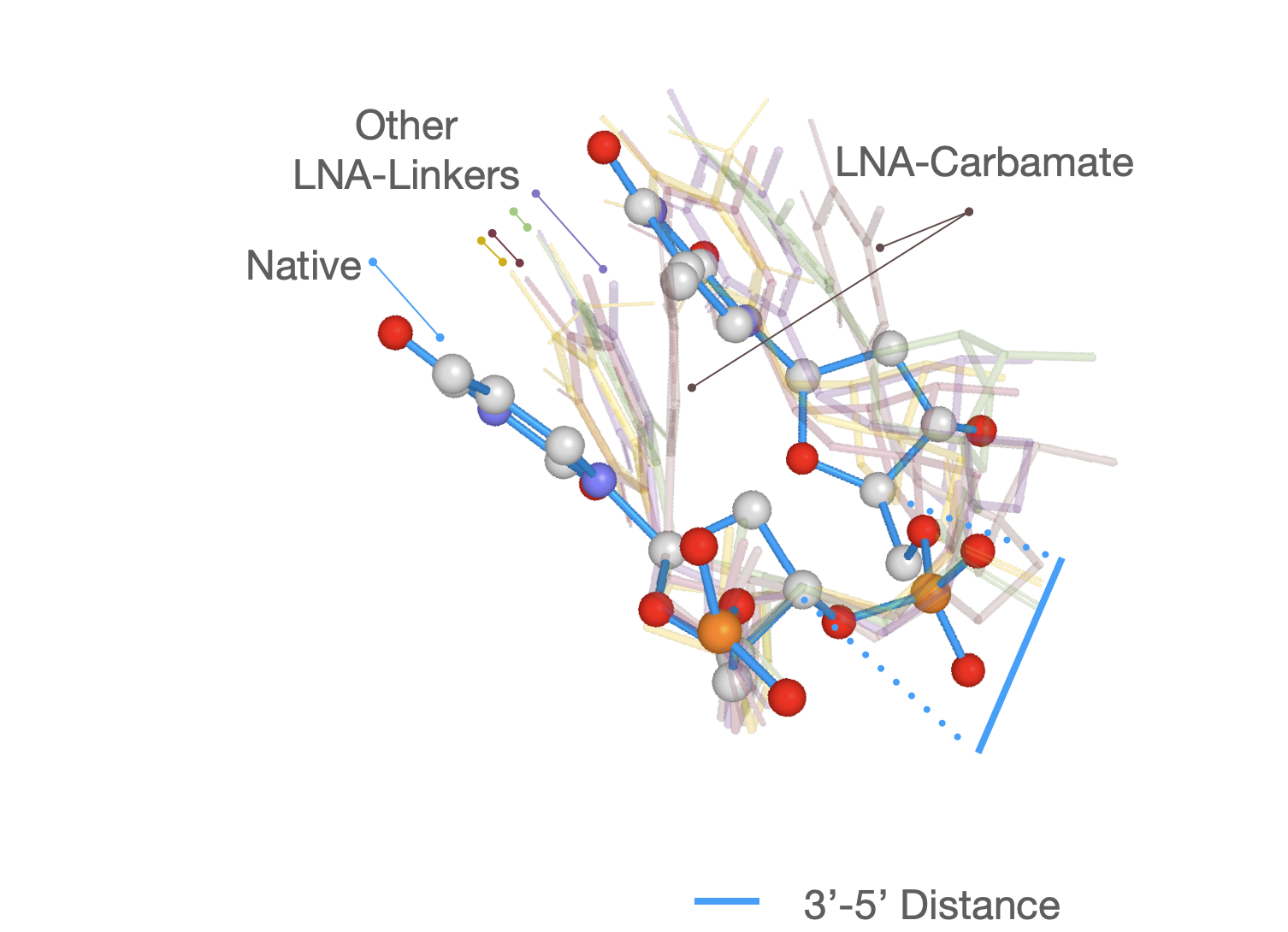


**Figure S23.** Overlayed structure of native (blue, stark) and all modified (transparent) thymidine residues, aligned at the 5’ sugar.

|  | Mean Distance (Å) | Distance Std. Deviation (Å) |
| --- | --- | --- |
| Native | 4.11 | 0.20 |
| LNA-PO | 3.85 | 0.17 |
| LNA-Amide | 4.50 | 0.16 |
| LNA-Sulfamate | 4.09 | 0.32 |
| LNA-Alkoxyamide | 4.78 | 0.22 |
| LNA-Carbamate | 4.54 | 0.12 |

**Table S3.** Key statistics about linker 3'-5' distances during the course of the combined 3 µs simulation time.

# Cell viability assays


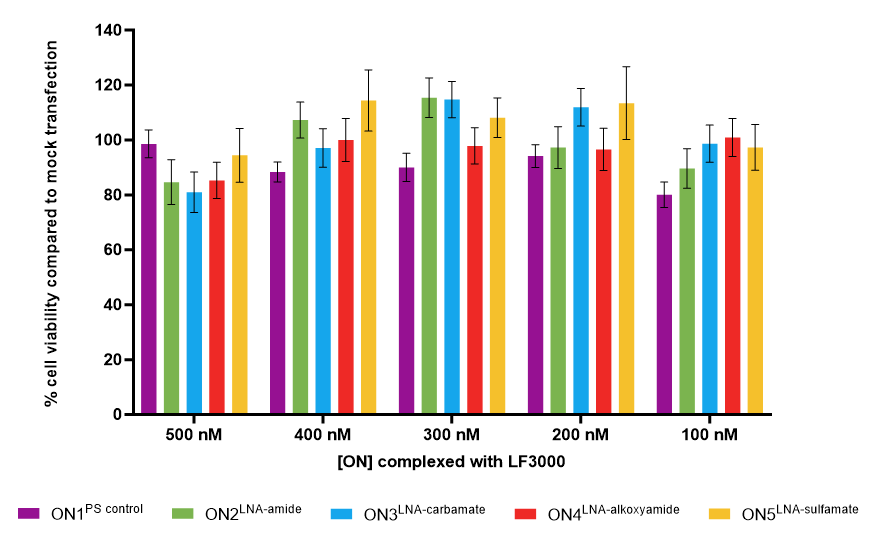


**Figure S24A.** Viability of the HeLa pLuc/705 cells following transfection with ON1-ON6 using LF3000 determined using WST-1 assay. The error bars are the means of technical replicates (n=3) plus/minus SD.

**Figure S24B.** *Transfection: Micrographs of HeLa pLuc/705 cells following transfection with ONs.* ONs were transfected into cells at the indicated concentrations using Lipofectamine 2000, and images were captured at the assay endpoint 48 h later. Scale bar = 1000 µm. Micrographs are representative of three images taken per condition. *Gymnosis: Micrographs of HeLa pLuc/705 cells following gymnosis with ONs.* ONs were applied to HeLa pLuc/705 cells at the indicated concentrations in the absence of a transfection reagent, and images were captured at the assay endpoint 72 h later. Scale bar = 1000 µm. Micrographs are representative of three images taken per condition.


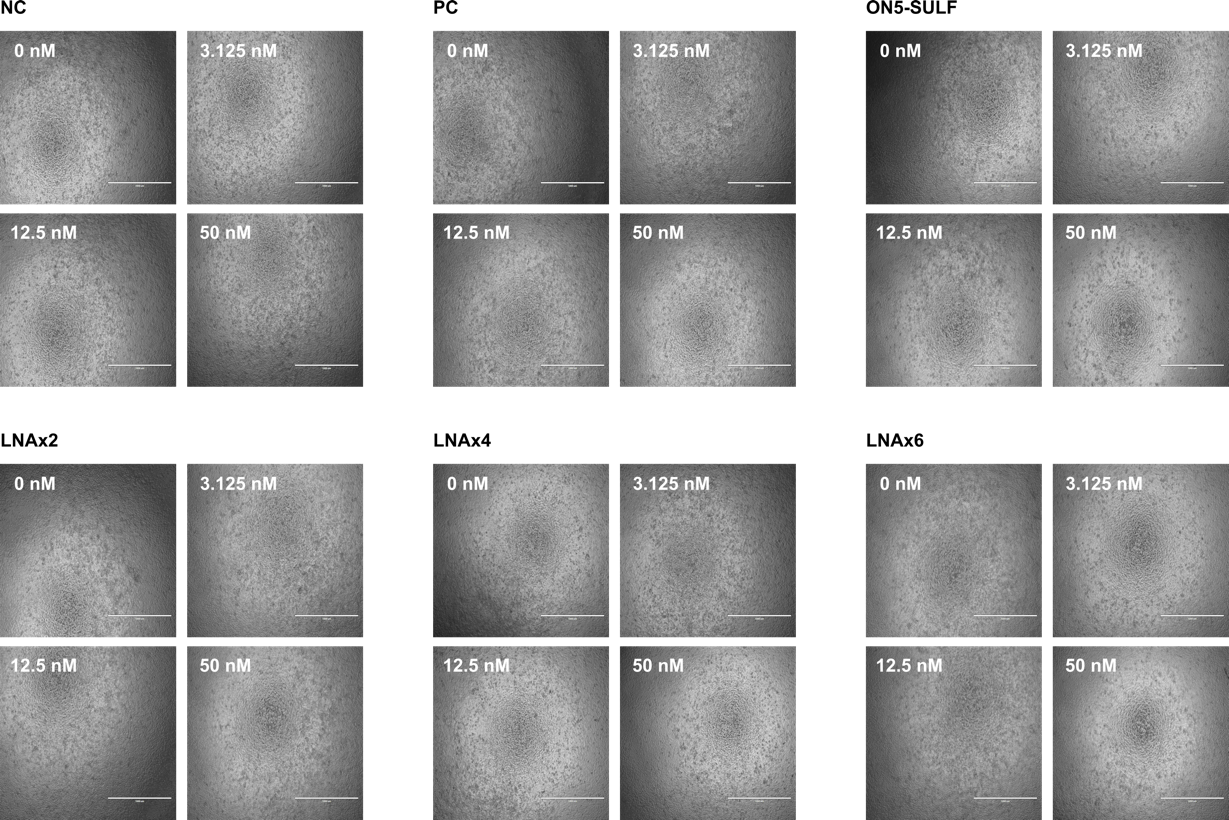
**Transfection**


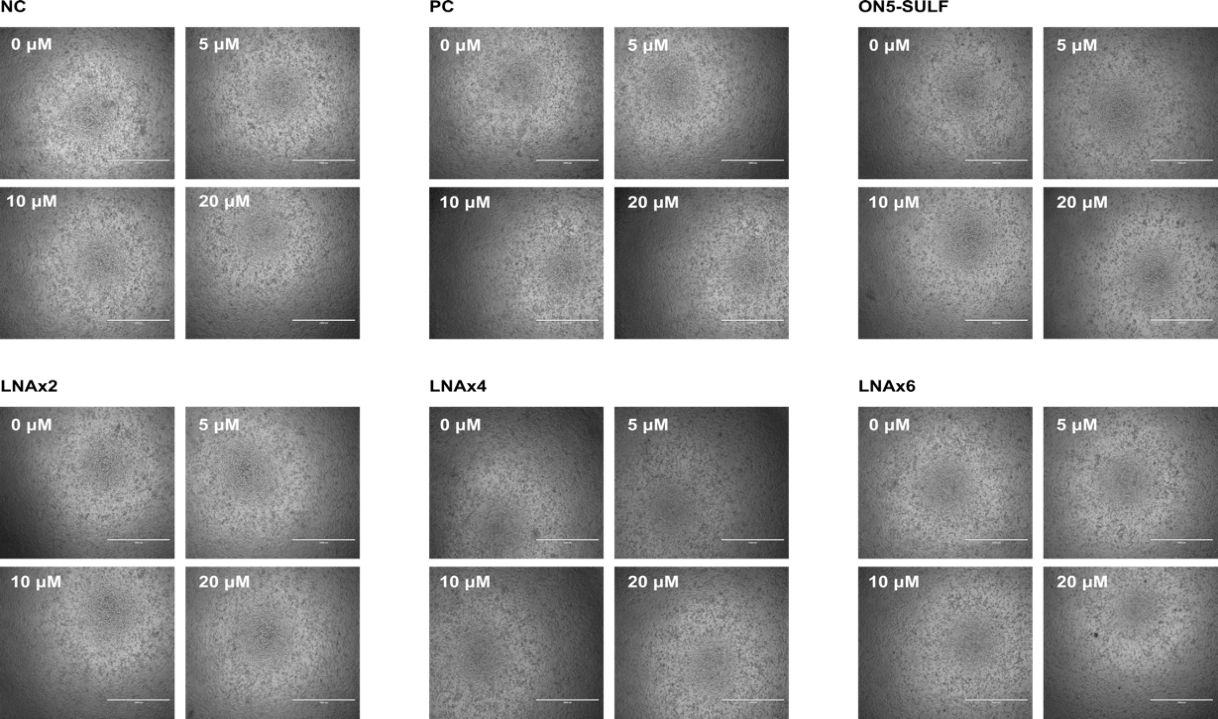


**Gymnosis**

# Luciferase assay transfection activity


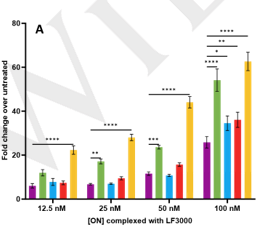


**Figure S25A.** Splice-switching activity of ON1-5 using the luciferase reporter assay (HeLa pLuc/705 cell line). Activity was measured as luminescence normalised first to protein quantity then to untreated cells with delivery by transfection with Lipofectamine 3000 (LF3000). Statistical significance was determined using a 2-way ANOVA test using ON1 control as the control within each concentration. *represents P ≤ 0.05, **represents P ≤ 0.01, ***represents P ≤ 0.001, ****represents P ≤ 0.0001. Statistical comparisons without labels are not significant (ns). All data are given as the means ±SE of distinct biological replicates (n=3); each biological replicate is the mean ±SD of technical replicates (n=3).

# Luciferase assay scramble activity

| 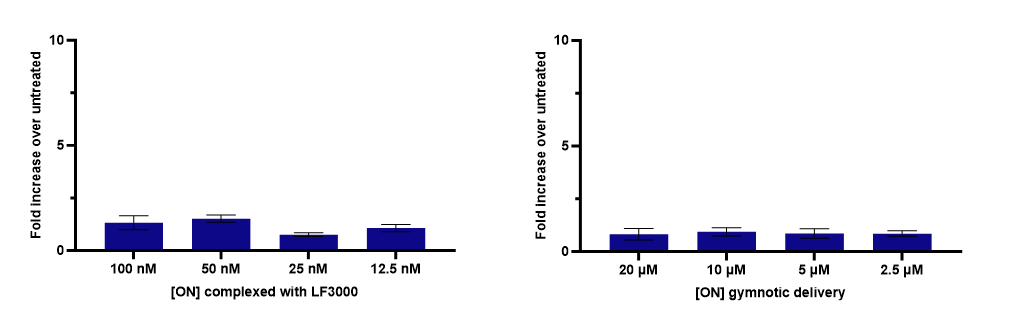 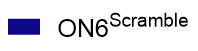 |
| --- |

**Figure S25B.** Luciferase activity of ON6^Scramble^. The activity is ~1-fold increase over untreated (equal to untreated) across all doses, when delivered via transfection (left) and gymnosis (right). The error bars are the means of technical replicates (n=3) plus/minus SD.

# Experimental

## Oligonucleotide synthesis

Automated solid-phase oligonucleotide synthesis was carried out on an Applied Biosystems 394 synthesiser using a standard phosphoramidite cycle of detritylation, coupling, capping, and sulfurisation. Synthesis was performed on a 1.0 µmol scale using 2'-OMe CPG solid supports (A(Bz), C(Ac)) and 2'OMe-phosphoramidites (U, A(Bz), C(Ac), G(dmf)) and LNA-T, all purchased from *Biosearch Technologies* (acquired from *LINK*)*.* Standard reagents such as Cap A, Cap B, BTT, and ACN were purchased from *Sigma Aldrich.* 3-ethoxy-1,2,4-dithiazoline-5-one (EDITH) was purchased from *Biosearch Technologies.* CH_2_Cl­_2_ (alcohol-free) was purchased from *Rathburn*. All phosphoramidites were dissolved in anhydrous ACN (0.1 M) immediately prior to use. 2’-OMe and LNA phosphoramidites were coupled for 5 min and custom dimer phosphoramidites (compounds 11, 13, 15, 17) were coupled for 10 min. Sulfurisation was carried out for 4 min using EDITH. Coupling efficiency was estimated using the trityl cation conductivity monitoring system built into the ABI synthesiser. The oligonucleotides were cleaved from the resin and deprotected by heating the resin in concentrated aqueous ammonia solution at 55 °C for 5 h.

Oligonucleotides were purified with the final 5’-DMTr group on using an Agilent 1260 Infinity system using a Clarity 5 µm Oligo-RP™ C18 Phenomenex column (250 × 10 mm). The gradient used was 20-80% Buffer B (60% ACN, 0.1 M TEAA) in Buffer A (0.1 M TEAA) over 30 min at a flow rate of 5 mL min^‑1^. Elution was monitored by UV absorption at 260 nm. Fractions containing product were combined, lyophilised, and the 5’-DMTr protecting group was removed by addition of 80% acetic acid (0.5 mL) for 30 min at rt, neutralised by the addition of 2 M TEAA buffer (0.5 mL), and the oligonucleotide was submitted to a simultaneous exchange to sodium salt and de-salting protocol. After loading the detritylated oligonucleotides onto a Luna 10 µm C8(2) 100 Å Phenomenex column (250 × 10 mm), the oligonucleotide was washed using 0.1 M NaOAc (2.5 column volumes), water (2.5 column volumes), and eluted in 50% ACN in water at a flow rate of 4 mL min^-1^. Elution was monitored by UV absorbance at 295 nm. Oligonucleotides were lyophilised and the concentration determined using a UV spectrometer or Nanodrop2000™ over three independent repeats, and ultrapure water was added to give 100 µM stock solutions.

ON7 (DNA reverse complement) and ON8 (RNA reverse complement) were purchased from *IDT Technologies.*

## Oligonucleotide characterisation

Oligonucleotide analysis

Oligonucleotides were characterised by negative-mode ultra-performance liquid chromatography (UPLC) mass spectrometry using a Waters Xevo G2-XS QT of mass spectrometer with an Acquity UPLC system, equipped with an Acquity UPLC oligonucleotide BEH C18 column (particle size: 1.7 μm; pore size: 130 Å; column dimensions: 2.1 mm x 50 mm). Data were analysed using Waters MassLynx software v 4.1 or Waters UNIFI Scientific Information System software.

UV melting experiments

UV melting experiments were performed using a Cary 4000 scan UV-vis spectrophotometer. 3 nmol of each oligonucleotide was dissolved in 1 mL of 10 mM phosphate buffer containing 0-200 mM NaCl at pH 7.0. The samples were first denatured by heating to 85 °C (10 °C/min) and then annealed by slowly cooling to 20 °C (1 °C/min). Six successive cycles of heating and cooling were performed at a gradient of 1 °C/min whilst recording the change in UV absorbance at 260 nm. The built-in Cary WinUV 3.0 software was then used to calculate the melting temperature from the first derivative of three melting curves, of which the average was taken as the *T*_m_.

CD spectroscopy

CD was recorded for duplexes formed from 3 µM ASO and 3 µM of a complementary DNA or RNA target in 10 mM phosphate buffer with 100 mM NaCl (pH 7.0). CD was performed on a Chirascan Plus spectrometer using a quartz cuvette with a pathlength of 1 mm. Scans were taken at 25 °C from 200–340 nm with 0.5 a step and 1.0 s time point intervals. The average of four scans was taken and smoothed to 20 points using a third order polynomial (Savitzky-Golay, Origin 2017). The spectra were then baseline corrected to the θ-value at 340 nm.

## Biological assays

Evaluation of stability in human serum

Five nmol of each oligonucleotide was dissolved in Dulbecco’s PBS (50 μL) and human serum (50 μL, Gibco, standard sterile-filtered) was added. The sample was mixed by pipetting and 20 μL of this solution was immediately removed, mixed with formamide (20 μL), snap frozen in liquid N_2_, and stored at −80 °C as a control (0 h). The remaining reaction mixtures were incubated at 37 °C and aliquots (20 μL) were taken 96 h later, mixed with formamide (20 μL), and samples were analysed by denaturing 20% polyacrylamide gel electrophoresis.

Cell culture

HeLa pLuc/705 cells were maintained in Dulbecco's Modified Eagle Medium (DMEM; Gibco, 31966) supplemented with 10% fetal bovine serum (FBS) and 1X Antibiotic-Antimycotic (Gibco, 15240) at 37 °C and 5% CO_2_ in a humidified incubator.

Transfection experiments

Method 1: HeLa pLuc/705 cells were seeded at a density of 10,000 cells/well in 100 µL DMEM supplemented with 10% FBS and 1X Antibiotic-Antimycotic in a 96-well plate (Greiner, 655098) and incubated for 16 h at 37 °C and 5% CO_2_ in a humidified incubator. Oligonucleotides in aqueous solution were diluted to 100 nM in 300 µL Opti-MEM (Gibco, 31985). Separately, 2.25 μL of Lipofectamine 2000 Transfection Reagent (Invitrogen, 11668) were diluted to a final volume of 300 μL in Opti-MEM and incubated for 5 min at room temperature. After 5 min, the diluted oligonucleotide and diluted transfection reagent were mixed and incubated for 20 min at room temperature to allow complex formation. After 20 min, the complexes were serially diluted in Opti-MEM to achieve final oligonucleotide concentrations of 50, 12.5, and 3.125 nM. At 16 h post-seeding, the complete media was aspirated from the cells and replaced with the diluted complexes or Opti-MEM alone (i.e., the 0 nM treatment or untreated cells). The cells were incubated for 4 h at 37 °C and 5% CO_2_ in a humidified incubator. At 4 h post-transfection, the complexes were aspirated from the cells and replaced with complete media, and the cells were incubated for a further 44 h at 37 °C and 5% CO_2_ in a humidified incubator.

Method 2: Cells were seeded at a density of 10,000 cells/well in 100 μL of growth media in 96 well plates 16 h before transfection to reach 70-80% cell confluency. Solutions of 2000 nM, 1000 nM, 500 nM, and 250 nM oligonucleotide in OptiMEM were made and diluted 1:1 v/v with lipofectamine 3000 in OptiMEM, following the manufacturer’s protocol, omitting P3000. The media in each well was replaced with 90 μL of growth media and after complexing for 10 min, 10 μL of the complexes were added to each well. The cells were then incubated at 37 °C in a humidified incubator with 5% CO_2_ for 24 h.

Gymnosis experiments

Oligonucleotides in aqueous solution were diluted to 40 µM in 400 µL Opti-MEM and then serially diluted in Opti-MEM to achieve oligonucleotide concentrations of 40, 20, and 10 µM. Then, 50 µL of diluted oligonucleotide or Opti-MEM alone (i.e., the 0 µM treatment or untreated cells) and 50 µL HeLa pLuc/705 cells in DMEM supplemented with 6% FBS and 2X Antibiotic-Antimycotic were added to each well of a 96-well plate (Greiner, 655098) to achieve final oligonucleotide concentrations of 20, 10, 5, and 0 µM and a seeding density of 10,000 cells/well in 100 µL DMEM supplemented with 3% FBS and 1X Antibiotic-Antimycotic. The cells were incubated for 72 h at 37 °C and 5% CO_2_ in a humidified incubator.

Luciferase assays

At the end of the transfection or gymnosis experiments described above, the complete media was aspirated from the cells, and the cells were washed with phosphate buffered saline (PBS; Gibco, 10010) or PBS followed by 0.1 mg/mL heparin (Merck, H3149) in PBS followed by PBS, respectively. Lysis was performed in 100 µL of Glo Lysis Buffer (Promega, E2661) for 10 min at room temperature with shaking. Then, 50 µL of lysate and 50 µL of Bright-Glo Reagent (Promega, E2620) were added to each well of a 96-well white plate (Greiner, 655075), and after 2 min, luminescence was measured using a CLARIOstar microplate reader (BMG Labtech, software version 5.21.R2). Total protein quantification was carried out using a detergent compatible (DC) Protein Assay (Bio-Rad, 5000111) according to the manufacturer’s instructions. Briefly, a bovine serum albumin (BSA) standard (Thermo Scientific, 23209) was prepared at a concentration range of 20-20,000 µg/mL. Then, 5 µL of lysate or BSA standard were treated with 15 µL of Reagent A’ and 120 µL of Reagent B and incubated for 20 min at room temperature. Absorbance at 750 nm was measured using a CLARIOstar microplate reader (BMG Labtech, software version 5.21.R2). Total protein quantities were calculated from the measured absorbances using the equation of the linear-fit standard curve in Microsoft Excel.

Both the transfection and gymnosis experiments described above were performed in biological triplicate, where each biological replicate was performed in technical triplicate. To calculate the final fold increase over untreated value shown in the plots, the three biological replicates were averaged. Data in the plots are means ± SEM for three biological replicates (*n* = 3). Statistical analyses were performed in GraphPad Prism 10 for macOS Version 10.3.1 (464).

WST-1 cell viability assay

The cell viability was evaluated using the WST-1 cell proliferation reagent (Roche) in accordance with the manufacturer’s guidelines. Briefly, cells were seeded and transfected using Lipofectamine 3000 as described above. The cells were then incubated for 24 h at 37 °C in a humidified incubator with 5% CO_2_ before WST-1 reagent (10 µL) was added to each well. The cells were returned to the incubator for 30 min. The absorbance at 440 nm was measured using a CLARIOstar microplate reader (BMG Labtech) and analysed with CLARIOstar software version 5.21.R2. Absorbance values were divided by the absorbance value for mock transfected cells to give a percentage viability compared to lipofectamine complexes without ASO.

## Chemistry

General experimental

Unless otherwise stated, reactions were performed in oven-dried glassware under an inert argon using anhydrous solvents. Anhydrous solvents were collected from an mBraun SPS-800 bench top solvent purification system, having passed through anhydrous alumina columns. Solvents for phosphitylation reaction were degassed by bubbling with argon before used and pyridine and CH_2_Cl_2_ were further purified by distillation over KOH or CaH respectively. All chemicals were used as obtained from commercial sources without further purification.

Thin layer chromatography (TLC) was performed using Merck pre-coated 0.23 mm thick plates of Kieselgel 60 F254 and visualised using UV (λ = 254 nm) or by staining with *p*-anisaldehyde. Column chromatography was carried out using Biotage Isolera automated chromatography instrument with Biotage Sfär silica duo cartridges or manually using Merck Geduran 60 Å (40‐63 micron) silica.

^1^H, ^13^C and ^31^P NMR spectra were recorded on a Bruker AVIIIHD 400, Bruker AVII 500 (with a 13C cryoprobe), or Bruker NEO 600 (with broadband helium cryoprobe) spectrometer operating at 400, 500 or 600 MHz respectively using an internal deuterium lock at ambient probe temperatures. ^1^H NMR chemical shifts (δ) are quoted to the nearest 0.01 ppm and are referenced relative to residual solvent peak. Coupling constants (*J*) are given to the nearest 0.1 Hz. The following abbreviations are used to indicate the multiplicity of signals: s = singlet, d = doublet, t = triplet, q = quartet, m = multiplet, and br = broad. Data is reported as follows: chemical shift (multiplicity, coupling constant(s), integration). ^13^C NMR chemical shifts (δ) are quoted to the nearest 0.1 ppm and are reference relative to the deuterated solvent peak. NMR assignments are supported by DEPT, COSY, HMQC, and HMBC where necessary. High resolution mass spectra (HRMS) were recorded on a Thermo Scientific Exactive Mass Spectrometer equipped with a Waters Equity autosampler and pump by the University of Oxford Chemistry Departmental Mass Spectrometry Service, and reported mass values are within ± 5 ppm mass units unless otherwise stated.

Synthesis of compounds

**5′-*O*-(Dimethoxytrityl)-LNA-amide-LNA-thymidine dimer (10)**

Compound **3** (450 mg, 0.732 mmol) was prepared according to the literature[^12^](#_ENREF_12) and DIPEA (0.256 ml, 1.47 mmol) in DMF (5 mL) were dried over 4Å molecular sieves (500 mg) at rt for 15 min. HATU (278 mg, 0.732 mmol) was added and the reaction was stirred at rt for 30 min before compound **1** (217 mg, 0.806 mmol) was added. The reaction was stirred at rt for 4 h and the solvent removed *in vacuo*. The residue was dissolved in EtOAc (10 mL), washed with sat. NaHCO_3_ (2 × 10 mL), dried over Na_2_SO_4_, and concentrated *in vacuo*. Purification via column chromatography (gradient elution, 0%→20% CH_3_OH in EtOAc with constant 1% pyridine) gave **10** as a white powder (488 mg, 77%); δ_H_ (500 MHz, CDCl_3_) 11.43 (s, 1H), 11.35 (s, 1H), 8.28 (t, J = 6.0 Hz, 1H), 7.55 (d, J = 1.3 Hz, 1H), 7.42 (dd, J = 8.5, 1.3 Hz, 1H), 7.25-7.45 (m, 9H), 6.89-6.92 (m, 4H), 5.53 (d, J = 3.9 Hz, 1H), 5.48 (s, 1H), 5.38 (s, 1H), 4.38 (s, 1H), 4.12 (s, 1H), 3.81 (d, J = 8.0 Hz, 1H), 3.76 (d, J = 3.6 Hz, 1H), 3.74 (d, J = 2.2 Hz, 6H), 3.69 (d, J = 8.5 Hz, 1H), 3.62 (d, J = 8.5 Hz, 1H), 3.56 (d, J = 8.0 Hz, 1H), 3.51 (t, J = 5.6 Hz, 2H), 3.48 (d, J = 11.5 Hz, 1H), 3.36 (d, J = 11.3 Hz, 1H), 2.48 (s, 1H), 2.20 (dd, J = 15.4, 8.6 Hz, 1H), 2.10 – 2.03 (m, 1H), 1.72 (d, J = 1.2 Hz, 3H), 1.55 (d, J = 1.1 Hz, 3H); δ_C_ (151 MHz, CDCl_3_) 170.4, 158.2, 144.6, 135.1, 135.1, 134.5, 134.2, 129.8, 129.8, 127.9, 127.7, 126.9, 113.3, 108.5, 108.5, 89.2, 87.3, 86.6, 86.4, 85.8, 79.8, 78.9, 71.4, 71.4, 70.0, 59.0, 55.0, 40.1, 35.3, 29.5, 12.3, 12.1; HRMS (ESI^+^) C_45_H_47_N_5_NaO_13_^+^ ([M+Na]^+^) requires 888.3063; found 888.3060.

**5′-*O*-(Dimethoxytrityl)-LNA-amide-LNA-thymidine dimer phosphoramidite (11)**

Chloro(diisopropylamino)-β cyanoethoxyphosphine (117 µL, 0.0523 mmol) was added to a solution of dimer **3** (302 mg, 0.349 mmol) and degassed DIPEA (152 µL, 0.872 mmol) in degassed THF (6.0 mL) under Ar and the reaction was stirred at rt for 2 h. The reaction was concentrated *in vacuo* and the residue was dissolved in CH_2_Cl_2_ (5 mL). The organic layer was washed with sat. aq. KCl (10 mL) and dried over Na_2_SO_4_ and concentrated *in vacuo*. The residue was dissolved in CH_2_Cl_2_ (0.5 mL) and precipitated from hexane (50 mL). The supernatant was discarded and the residue was purified via column chromatography (eluent EtOAc with 1% pyridine) to give phosphoramidite **11** as a white solid (195 mg, 52%); δ_P_ (162 MHz, CD_3_CN) 149.2, 148.9; HRMS (ESI^+^) C_55_H_68_N_7_NaO_14_P^+^ ([M+Na]^+^) requires 1104.4460; found 1104.4458.

**5′-*O*-(Dimethoxytrityl)-LNA-carbamate-LNA-thymidine phosphoramidite dimer (12)**

Dimer **12** was prepared as previously reported. Characterisation data are consistent with the literature.[^24^](#_ENREF_24)

**5′-*O*-(Dimethoxytrityl)-LNA-carbamate-LNA-thymidine phosphoramidite dimer (13)**

Phosphoramidite **13** was prepared as previously reported. Characterisation data are consistent with the literature.[^24^](#_ENREF_24)

***N*^3^-Benzoyl-5′-*O*-dimethoxytrityl-LNA-thymidine (5)**

BSA (*N*,*O*-bis(trimethylsilyl)acetamide, 1.37 mL, 5.59 mmol) was added to a solution of compound **2** (1.60 g, 2.79 mmol) in MeCN (45.0 mL) under Ar and the reaction was heated at 80 °C for 1 h. The reaction was cooled to rt, and Et_3_N (780 µL, 5.59 mmol) and BzCl (422 µL, 3.63 mmol) were added and the reaction was stirred at rt for 16 h. TBAF (1 M in THF, 4.19 mL, 4.19 mL) was added and the reaction was stirred at rt for 5 h. The reaction was concentrated *in vacuo* and the residue was partitioned between EtOAc and sat. NaHCO_3_ (v/v, 1:1, 400 mL). The organic layer was separated, dried over Na_2_SO_4_ and concentrated *in vacuo*. Purification via column chromatography (gradient elution, 0%→55% EtOAc in petroleum ether with constant 1% TEA gave compound **5** as a white solid (1.40 g, 74%); δ_H_ (600 MHz, Acetone-*d*_6_) 8.08 – 8.03 (m, 2H), 7.89 (d, J = 1.4 Hz, 1H), 7.79 – 7.73 (m, 1H), 7.61 – 7.55 (m, 4H), 7.47 – 7.40 (m, 4H), 7.40 – 7.33 (m, 2H), 7.32 – 7.26 (m, 1H), 6.97 – 6.91 (m, 4H), 5.61 (s, 1H), 4.54 (s, 1H), 4.38 (s, 1H), 3.95 (d, J = 7.9 Hz, 1H), 3.82 (s, 6H), 3.80 (s, 1H), 3.66 (d, J = 10.9 Hz, 1H), 3.56 (d, J = 11.0 Hz, 1H), 1.70 (d, J = 1.1 Hz, 3H); δ_C_ (101 MHz, Acetone-*d*_6_) 169.5, 162.7, 158.9, 149.8, 148.9, 145.1, 135.8, 135.6, 135.0, 132.1, 130.4, 130.2, 130.1, 129.2, 128.1, 127.9, 126.9, 123.7, 113.2, 109.2, 88.2, 87.5, 86.4, 79.4, 71.6, 70.2, 59.7, 58.8, 54.7, 12.0; HRMS (ESI^+^) C_39_H_36_N_2_NaO_9_^+^ ([M+Na]^+^) requires 699.2319; found 699.2321.

***N*^3^-Benzoyl—5′-*O*-dimethoxytrityl-LNA-thymidine 3’*O*-methyl ester (6)**

NaH (60% in mineral oil, 156 mg, 3.90 mmol) was added to a solution of compound **5** (1.20 g, 1.77 mmol) in DMF (2 mL) at 0 °C under Ar and the reaction was stirred at rt for 1 h. Methyl bromoacetate (202 µL, 2.13 mmol) was added and the reaction was stirred at rt for 16 h. The reaction was concentrated in vacuo and the residue was partitioned between EtOAc and brine (1:1, v/v, 300 mL). The organic layer was dried over Na_2_SO_4_ and concentrated in vacuo. Purification via column chromatography (gradient elution, 20%→50% EtOAc in pet. ether 40-60 °C with a constant additive of 1% pyridine) gave ester **6** as a white solid (0.90 g, 70%); δ_H_ (400 MHz, Acetone) 7.91 – 7.86 (m, 2H), 7.75 (d, J = 1.3 Hz, 1H), 7.65 – 7.59 (m, 1H), 7.48 – 7.41 (m, 4H), 7.35 – 7.29 (m, 4H), 7.26 – 7.20 (m, 2H), 7.19 – 7.11 (m, 1H), 6.83 – 6.78 (m, 4H), 5.49 (s, 1H), 4.57 (s, 1H), 4.50 (s, 1H), 4.14 (d, J = 2.6 Hz, 2H), 3.77 (d, J = 7.9 Hz, 1H), 3.70 – 3.63 (m, 7H), 3.53 (s, 3H), 3.46 (d, J = 1.7 Hz, 2H), 1.54 (d, J = 1.2 Hz, 3H); δ_C_ (151 MHz, Acetone) 169.8, 169.4, 162.7, 158.9, 158.9, 148.9, 145.0, 135.6, 135.5, 135.0, 134.9, 132.1, 130.3, 130.2, 130.1, 130.1, 129.2, 128.1, 128.0, 128.0, 126.9, 113.2, 109.2, 87.6, 86.5, 77.3, 77.1, 71.9, 66.7, 58.4, 54.7, 51.1, 12.1; HRMS (ESI^+^) C_42_H_40_N_2_NaO_11_^+^ ([M+Na]^+^) requires 771.2530; found 771.2252.

**5′-*O*-dimethoxytrityl-LNA-thymidine-3’*O*-ethanoic acid (7)**

NaOH (2 M in H_2_O, 5.08 mL, 2.54 mmol) was added to a solution of compound **6** (950 mg, 1.27 mmol) in THF/MeOH (1:1, v/v, 10 mL) and the reaction was stirred at rt for 16 h. The reaction was concentrated *in vacuo* and purification via reversed phase chromatography (gradient elution, 0%→100% MeCN in H_2_O) gave acid **7** as a white solid (528 mg, 66%); δ_H_ (400 MHz, DMSO-*d*_6_) 11.42 (s, 1H), 7.58 (d, J = 1.5 Hz, 1H), 7.45 (d, J = 7.3 Hz, 2H), 7.36 – 7.28 (m, 6H), 7.25 (dd, J = 8.3, 6.2 Hz, 1H), 6.95 – 6.88 (m, 4H), 5.47 (s, 1H), 4.50 (s, 1H), 4.32 (s, 1H), 3.74 (d, J = 3.4 Hz, 10H), 3.46 – 3.33 (m, 2H), 1.58 (d, J = 1.0 Hz, 3H); δ_C_ (151 MHz, DMSO-*d*_6_) 172.1, 164.3, 158.6, 150.4, 145.1, 135.8, 135.6, 134.8, 130.4, 130.3, 130.2, 128.4, 128.2, 127.3, 113.8, 113.5, 109.0, 87.0, 86.2, 77.6, 76.9, 72.2, 69.8, 59.2, 55.5, 46.0, 13.0; HRMS (ESI^+^) C_34_H_34_N_2_NaO_10_^+^ ([M+Na]^+^) requires 653.2111; found 653.2099.

**5′-*O*-(Dimethoxytrityl)-LNA-methoxyacetamide-LNA-thymidine dimer (14)**

HATU (844 mg, 2.22 mmol) was added to a solution of acid **7** (700 mg, 1.11 mmol), compound **1** (358 mg, 1.33 mmol) and Et_3_N (460 µL, 3.33 mmol) in DMF (3 mL) and the reaction was stirred at rt for 3 h. The reaction was concentrated *in vacuo* and purification via reversed phase chromatography (gradient elution, 0%→100% MeCN in H_2_O) gave dimer **14** as a white solid (700 mg, 72%); δ_H_ (600 MHz, Acetone) 10.04 (s, 2H, NH), 7.78 (q, J = 1.2 Hz, 1H), 7.77 – 7.73 (m, 1H), 7.59 – 7.55 (m, 2H), 7.45 – 7.40 (m, 5H), 7.37 (dd, J = 8.5, 7.0 Hz, 2H), 7.31 – 7.26 (m, 1H), 6.96 – 6.91 (m, 4H), 5.64 (s, 1H), 5.49 (s, 1H), 4.71 (s, 1H), 4.56 (s, 1H), 4.32 (d, J = 15.5 Hz, 2H), 4.20 (d, J = 15.5 Hz, 1H), 4.02 (dd, J = 8.0, 5.2 Hz, 2H), 3.89 – 3.83 (m, 2H), 3.83 – 3.80 (m, 6H), 3.78 (d, J = 6.1 Hz, 1H), 3.71 – 3.66 (m, 2H), 3.60 – 3.56 (m, 1H), 3.54 (d, J = 11.2 Hz, 1H), 1.80 (d, J = 1.2 Hz, 3H), 1.69 (d, J = 1.3 Hz, 3H); δ_C_ (151 MHz, Acetone) 170.4, 163.4, 163.3, 158.9, 149.9, 149.9, 144.9, 135.5, 135.4, 134.1, 134.0, 130.0, 130.0, 127.9, 126.9, 113.2, 113.2, 109.2, 109.1, 87.9, 87.3, 87.3, 87.2, 86.5, 79.0, 77.8, 77.1, 71.9, 71.6, 71.0, 69.2, 58.5, 54.6, 35.6, 12.1, 11.8; HRMS (ESI^+^) C_45_H_47_N_5_O_14_Na^+^ ([M+Na]^+^) requires 904.3017, found 904.3012.

**5′-*O*-(Dimethoxytrityl)-LNA-methoxyacetamide-LNA-thymidine dimer phosphoramidite (15)**

Chloro(diisopropylamino)-β cyanoethoxyphosphine (152 µL, 680 µmol) was added to a solution of dimer **14** (600 mg, 0.680 mmol) and Et_3_N (285 µL, 2.00 mmol) in CH_2_Cl_2_ (5.0 mL) under Ar and the reaction was stirred at rt for 3 h. The reaction was quenched by addition of sat. aq. KCl (5 mL) and the organic layer was dried over Na_2_SO_4_ and concentrated *in vacuo*. Purification via column chromatography (eluent EtOAc with 1% pyridine) gave phosphoramidite **15** as a white solid (470 mg, 65%); δ_P_ (162 MHz, CD_3_CN) 149.0, 148.6, HRMS (ESI^+^) C_54_H_64_N_7_NaO_15_P^+^ ([M+Na]^+^) requires 1104.4096; found 1104.6014.

**3’-TBS-5’-amino-LNA-thymidine (8)**

Compound **8** was synthesised by the alternative method below to that reported previously. Characterisation data are consistent with the literature.[^25^](#_ENREF_25) [^12^](#_ENREF_12)

Compound 1 (500 mg, 1.86 mmol) was dissolved in anhydrous pyridine (20 mL) and 4-methoxytriphenylmethyl chloride (MMTrCl) (805 mg, 2.61 mmol) was added portion-wise over 1 h. The reaction was stirred for 3 h at rt and the solvent was removed under reduced pressure, and re-dissolved in toluene (20 mL) which was removed under reduced pressure (3x). The residue was dissolved in DMF (20 mL) and imidazole (252 mg, 3.72 mmol) and TBSCl (558 mg, 3.72 mmol) were added and the reaction was stirred at rt for 16 h. The solvent was removed under reduced pressure and the crude dissolved in 3% TCA/CH_2_Cl_2_ (10 mL) and stirred at rt for 1 h. The reaction was purified by column chromatography (0-70% EtOAc/Pet. Ether) to give 8 (464 mg, 1.21 mmol, 65%) as a white foam.

**5′-(4-Nitrophenyl sulfamate)-amino-LNA-thymidine (9)**

Compound **9** was synthesised as previously reported. Characterisation data are consistent with the literature.[^25^](#_ENREF_25)

**5′-*O*-(Dimethoxytrityl)-LNA-sulfamate-LNA-thymidine dimer (16)**

Compound **16** was synthesised as previously reported. Characterisation data are consistent with the literature.[^25^](#_ENREF_25)

**5′-*O*-(Dimethoxytrityl)-LNA-sulfamate-LNA-thymidine phosphoramidite dimer (17)**

Compound **17** was synthesised as previously reported. Characterisation data are consistent with the literature.[^25^](#_ENREF_25)

#

# NMR spectra of compounds

**Figure S26.** ^1^H NMR (500 MHz, DMSO) spectrum of compound **10**.

**Figure S27.** ^13^C NMR (151 MHz, DMSO) spectrum of compound **10**.

**Figure S28.** ^31^P NMR (203 MHz, CDCl_3_) spectrum of compound **11**. **Figure S29.** ^31^P NMR (162 MHz, DMSO) spectrum of compound **13**. Data are consistent with the literature.[^24^](#_ENREF_24)

**Figure S30.** ^1^H NMR (400 MHz, acetone-*d*_6_) spectrum of compound **5**.

**Figure S31.** ^13^C NMR (101 MHz, acetone-*d*_6_) spectrum of compound **5**.

**Figure S32.** ^1^H NMR (400 MHz, acetone-*d*_6_) spectrum of compound **6**.

**Figure S33.** ^13^C NMR (101 MHz, acetone-*d*_6_) spectrum of compound **6**.

**Figure S34.** ^1^H NMR (600 MHz, DMSO-*d*_6_) spectrum of compound **7**.

**Figure S35.** ^13^C NMR (151 MHz, DMSO-*d*_6_) spectrum of compound **7**.

**Figure S36.** ^1^H NMR (400 MHz, acetone-*d*_6_) spectrum of compound **14**.

**Figure S37.** ^13^C NMR (101 MHz, acetone-*d*_6_) spectrum of compound **14**.

**Figure S38.** ^31^P NMR (162 MHz, CD_3_CN) spectrum of compound **15**.

**Figure S39.** ^1^H NMR (400 MHz, CDCl_3_) spectrum of compound **9**.

**Figure S40.** ^1^H NMR (101 MHz, CDCl_3_) spectrum of compound **9**.

**Figure S41.** ^1^H NMR (400 MHz, DMSO-*d*_6_) spectrum of compound **16**.

**Figure S42.** ^13^C NMR (151 MHz, DMSO-*d*_6_) spectrum of compound **16**.

**Figure S43.** ^31^P NMR (162 MHz, CD_3_CN) spectrum of compound **17**.

# UPLC and MS analysis of oligonucleotides with LNA-neutral linkages

**Figure S44.** Reverse-phase UPLC (UV absorbance at 260 nm vs time in min) and mass spectrum (ES^-^) of ON1^PS Control^ after HPLC purification. Required 6097.9 Da, found 6098.6 Da. y-axis = relative intensity (%), x-axis = mass in Da.

**Figure S45.** Reverse-phase UPLC (UV absorbance at 260 nm vs time in min) and mass spectrum (ES^-^) of ON2^2’OMe/LNA-amide^ after HPLC purification. Required 6036.5 Da, found 6036.8 Da. y-axis = relative intensity (%), x-axis = mass in Da.

**Figure S46.** Reverse-phase UPLC (UV absorbance at 260 nm vs time in min) and mass spectrum (ES^-^) of ON3^2’OMe/LNA-carbamate^ after HPLC purification. Required 6040.4 Da, found 6040.7 Da. y-axis = relative intensity (%), x-axis = mass in Da.

**Figure S47.** Reverse-phase UPLC (UV absorbance at 260 nm vs time in min) and mass spectrum (ES^-^) of ON4^2’OMe/LNA-alkoxyamide^ after HPLC purification. Required 6070.1 Da, found 6070.0 Da. y-axis = relative intensity (%), x-axis = mass in Da.

**Figure S48.** Reverse-phase UPLC (UV absorbance at 260 nm vs time in min) and mass spectrum (ES^-^) of ON5^2’OMe/LNA-sulfamate^ after HPLC purification. Required 6112.4 Da, found 6112.7 Da. y-axis = relative intensity (%), x-axis = mass in Da.


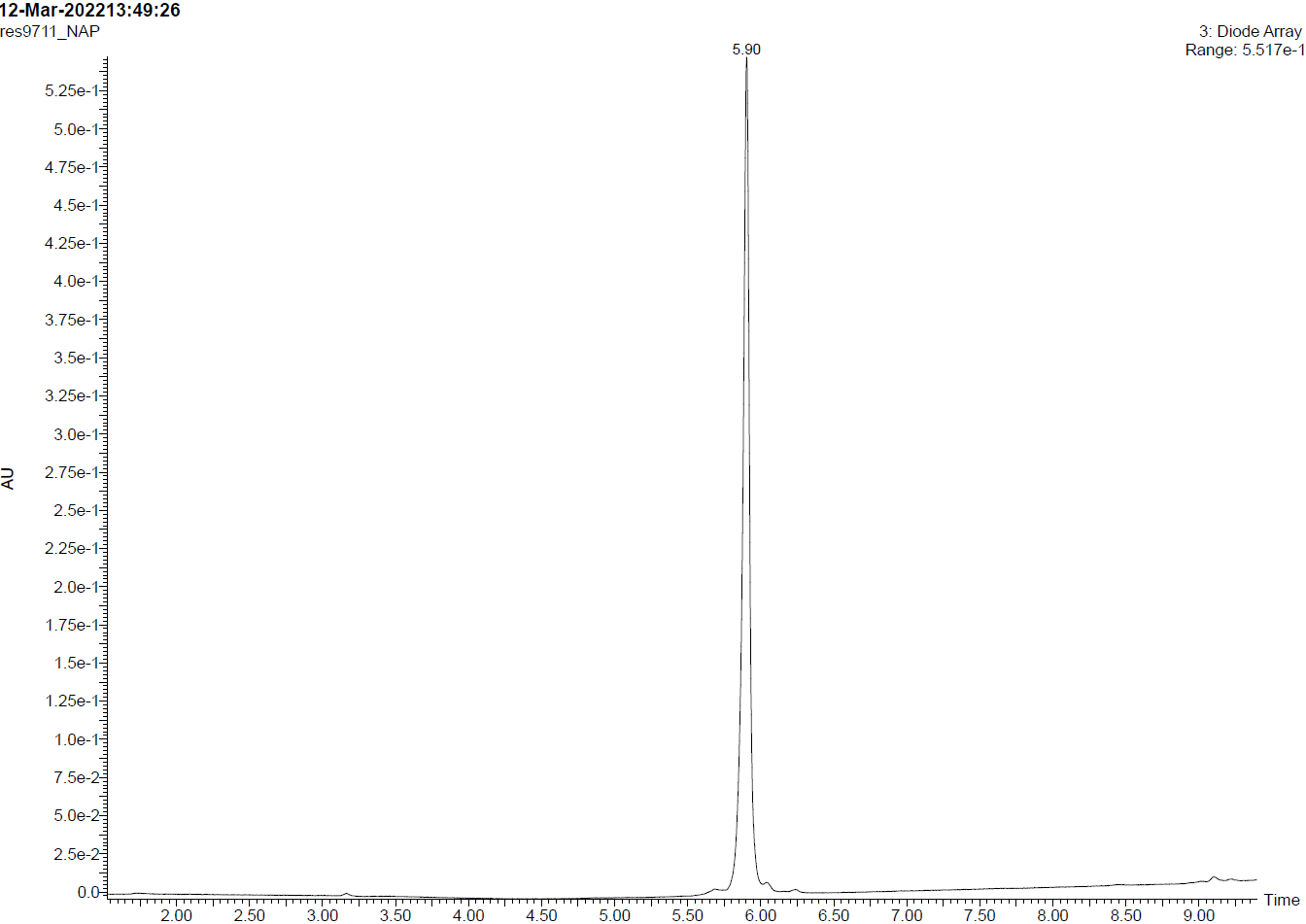


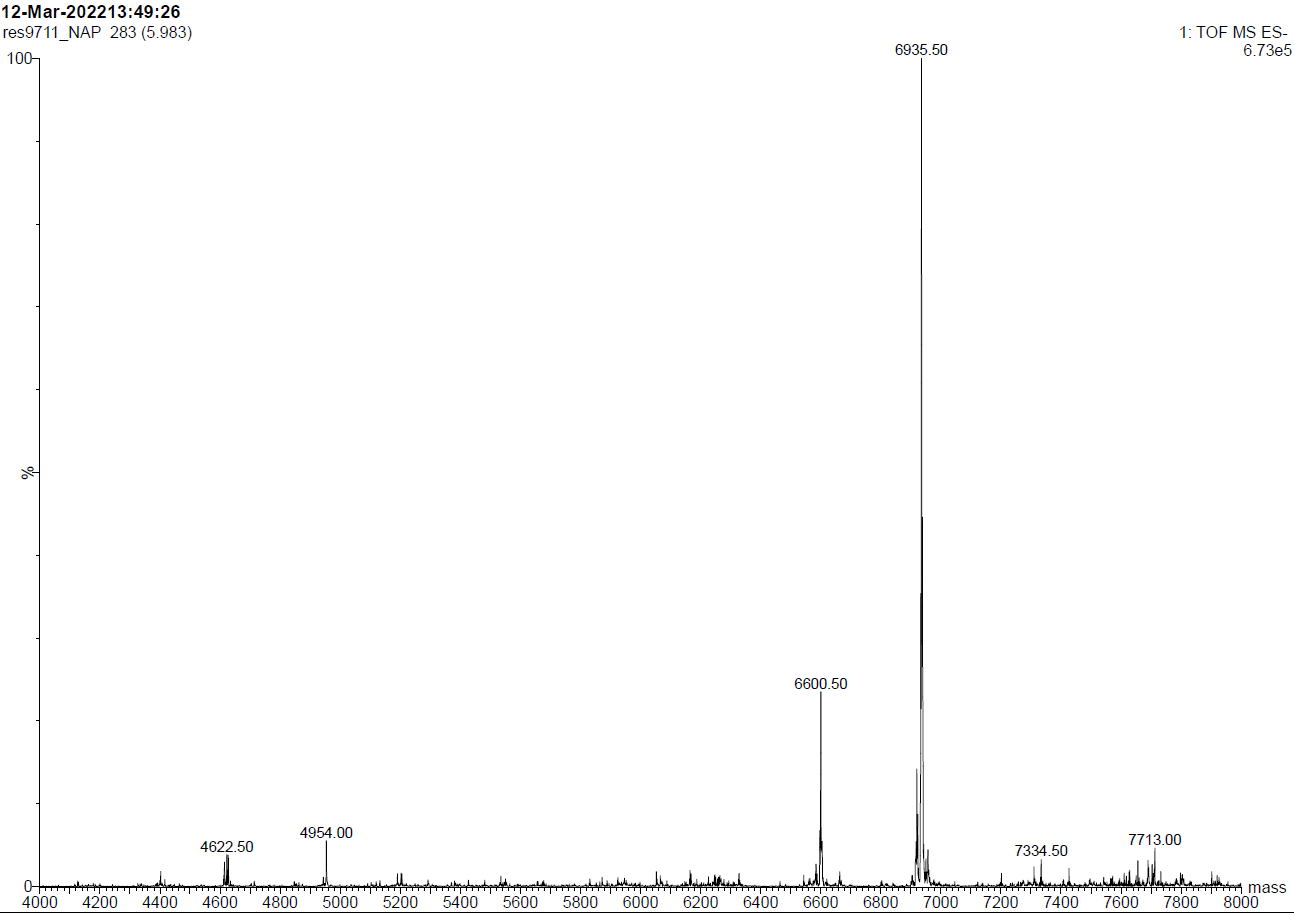


**Figure S49.** Reverse-phase UPLC (UV absorbance at 260 nm vs time in min) and mass spectrum (ES^-^) of ON6^PS Scramble^ after HPLC purification. Required 6933.9 Da, found 6935.9 Da. y-axis = relative intensity (%), x-axis = mass in Da.

**Figure S50.** Reverse-phase UPLC (UV absorbance at 260 nm vs time in min) and mass spectrum (ES^-^) of ON8^RNA-reverse_complement^ after HPLC purification. Required 5859.6 Da, found 5859.5 Da. y-axis = relative intensity (%), x-axis = mass in Da. Purchased from IDT Technologies.

# References

1 Cieplak, P., Cornell, W. D., Bayly, C. & Kollman, P. A. Application of the Multimolecule and Multiconformational Resp Methodology to Biopolymers - Charge Derivation for DNA, Rna, and Proteins. *J. Comput. Chem.* **16**, 1357-1377 (1995). <https://doi.org/DOI> 10.1002/jcc.540161106

2 Singh, U. C. & Kollman, P. A. An Approach to Computing Electrostatic Charges for Molecules. *J. Comput. Chem.* **5**, 129-145 (1984). <https://doi.org/DOI> 10.1002/jcc.540050204

3 Neese, F. Software update: The ORCA program system-Version 5.0. *Wires Comput. Mol. Sci.* **12** (2022). <https://doi.org/10.1002/wcms.1606>

4 Neese, F. The ORCA program system. *Wires Comput. Mol. Sci.* **2**, 73-78 (2012). <https://doi.org/10.1002/wcms.81>

5 Lin, Y. S., Li, G. D., Mao, S. P. & Chai, J. D. Long-Range Corrected Hybrid Density Functionals with Improved Dispersion Corrections. *J. Chem. Theory Comput.* **9**, 263-272 (2013). <https://doi.org/10.1021/ct300715s>

6 Weigend, F. & Ahlrichs, R. Balanced basis sets of split valence, triple zeta valence and quadruple zeta valence quality for H to Rn: Design and assessment of accuracy. *Phys. Chem. Chem. Phys.* **7**, 3297-3305 (2005). <https://doi.org/DOI> 10.1039/b508541a

7 Wang, J. M., Cieplak, P. & Kollman, P. A. How well does a restrained electrostatic potential (RESP) model perform in calculating conformational energies of organic and biological molecules? *J. Comput. Chem.* **21**, 1049-1074 (2000). <https://doi.org/Doi> 10.1002/1096-987x(200009)21:12<1049::Aid-Jcc3>3.3.Co;2-6

8 Condon, D. E. *et al.* Optimization of an AMBER Force Field for the Artificial Nucleic Acid, LNA, and Benchmarking with NMR of L(CAAU). *J Phys Chem B* **118**, 1216-1228 (2014). <https://doi.org/10.1021/jp408909t>

9 Wang, J., Wang, W., Kollman, P. A. & Case, D. A. Automatic atom type and bond type perception in molecular mechanical calculations. *J. Mol. Graph. Model.* **25**, 247-260 (2006). <https://doi.org/10.1016/j.jmgm.2005.12.005>

10 Wang, J., Wolf, R. M., Caldwell, J. W., Kollman, P. A. & Case, D. A. Development and testing of a general amber force field. *J. Comput. Chem.* **25**, 1157-1174 (2004). <https://doi.org/10.1002/jcc.20035>

11 Shirts, M. R. *et al.* Lessons learned from comparing molecular dynamics engines on the SAMPL5 dataset. *J Comput. Aided Mol. Des.* **31**, 147-161 (2017). <https://doi.org/10.1007/s10822-016-9977-1>

12 Baker, Y. R. *et al.* An LNA-amide modification that enhances the cell uptake and activity of phosphorothioate exon-skipping oligonucleotides. *Nat. Comm.* **13**, 4036 (2022). <https://doi.org/10.1038/s41467-022-31636-2>

13 GROMACS 2021.3 (Zenodo, 2021).

14 Price, D. J. & Brooks, C. L., 3rd. A modified TIP3P water potential for simulation with Ewald summation. *J. Chem. Phys.* **121**, 10096-10103 (2004). <https://doi.org/10.1063/1.1808117>

15 Galindo-Murillo, R., Roe, D. R. & Cheatham, T. E., 3rd. Convergence and reproducibility in molecular dynamics simulations of the DNA duplex d(GCACGAACGAACGAACGC). *Biochim. Biophys. Acta* **1850**, 1041-1058 (2015). <https://doi.org/10.1016/j.bbagen.2014.09.007>

16 Darden, T., York, D. & Pedersen, L. Particle Mesh Ewald - an N.Log(N) Method for Ewald Sums in Large Systems. *J. Chem. Phys.* **98**, 10089-10092 (1993). <https://doi.org/Doi> 10.1063/1.464397

17 Bussi, G., Donadio, D. & Parrinello, M. Canonical sampling through velocity rescaling. *J. Chem. Phys.* **126** (2007). <https://doi.org/10.1063/1.2408420>

18 Bernetti, M. & Bussi, G. Pressure control using stochastic cell rescaling. *J. Chem. Phys.* **153** (2020). <https://doi.org/10.1063/5.0020514>

19 Hess, B., Bekker, H., Berendsen, H. J. C. & Fraaije, J. G. E. M. LINCS: A linear constraint solver for molecular simulations. *J. Comput. Chem.* **18**, 1463-1472 (1997). <https://doi.org/Doi> 10.1002/(Sici)1096-987x(199709)18:12<1463::Aid-Jcc4>3.0.Co;2-H

20 Pettersen, E. F. *et al.* UCSF chimera - A visualization system for exploratory research and analysis. *J. Comput. Chem.* **25**, 1605-1612 (2004). <https://doi.org/10.1002/jcc.20084>

21 The PyMOL Molecular Graphics System v. 1.8 (2015).

22 Roe, D. R. & Cheatham, T. E. PTRAJ and CPPTRAJ: Software for Processing and Analysis of Molecular Dynamics Trajectory Data. *J Chem Theory Comput* **9**, 3084-3095 (2013). <https://doi.org/10.1021/ct400341p>

23 Case, D. A. *et al.* AmberTools. *J. Chem. Inf. Model.* **63**, 6183-6191 (2023). <https://doi.org/10.1021/acs.jcim.3c01153>

24 Thorpe, C., Epple, S., Woods, B., El-Sagheer, A. H. & Brown, T. Synthesis and biophysical properties of carbamate-locked nucleic acid (LNA) oligonucleotides with potential antisense applications. *OBC* **17**, 5341-5348 (2019). <https://doi.org/10.1039/C9OB00691E>

25 Zengin Kurt, B., Dhara, D., El-Sagheer, A. H. & Brown, T. Synthesis and Properties of Oligonucleotides Containing LNA-Sulfamate and Sulfamide Backbone Linkages. *Org. Lett.* **26**, 4137-4141 (2024). <https://doi.org/10.1021/acs.orglett.4c01232>
